# Programmable nanomedicine via bioorthogonal molecular engineering

**DOI:** 10.1186/s40580-026-00568-8

**Published:** 2026-07-29

**Authors:** Sunwoo Im, Seyoung Koo, Jong Seung Kim

**Affiliations:** 1 Department of Applied Chemistry, Center for Bionano Intelligence Education and Research, Hanyang University, Ansan, 15588 Korea; 2 Department of Energy and Bio Sciences, Hanyang University, Ansan, 15588 Korea; 3 Department of Chemistry, Korea University, Seoul, 02841 Korea; 4 National Research Laboratory for Convergence Degradation Biology, Korea University, Seoul, 02841 Korea

**Keywords:** Bioorthogonal chemistry, Nanomedicine, Post-functionalization, Prodrug activation, In situ assembly, Diagnostic imaging, Targeted therapy

## Abstract

Bioorthogonal chemistry provides a versatile strategy for programming nanomedicines beyond static carrier design. It enables post-synthetic installation of functional modules on nanoparticle surfaces, spatiotemporal control of payload activation and release, and higher-order functions arising from nanoparticle aggregation or nanoparticle–cell conjugation in diseased tissues. These capabilities allow nanomedicines to be activated more selectively in space and time and to generate functions that are difficult to achieve with conventional formulations. This review summarizes recent advances in multiscale bioorthogonal engineering for programmable nanomedicines, focusing on surface functionalization, molecular control of drug release, and interparticle or cell-associated assembly, and discusses challenges and future directions.

**Graphical abstract:**

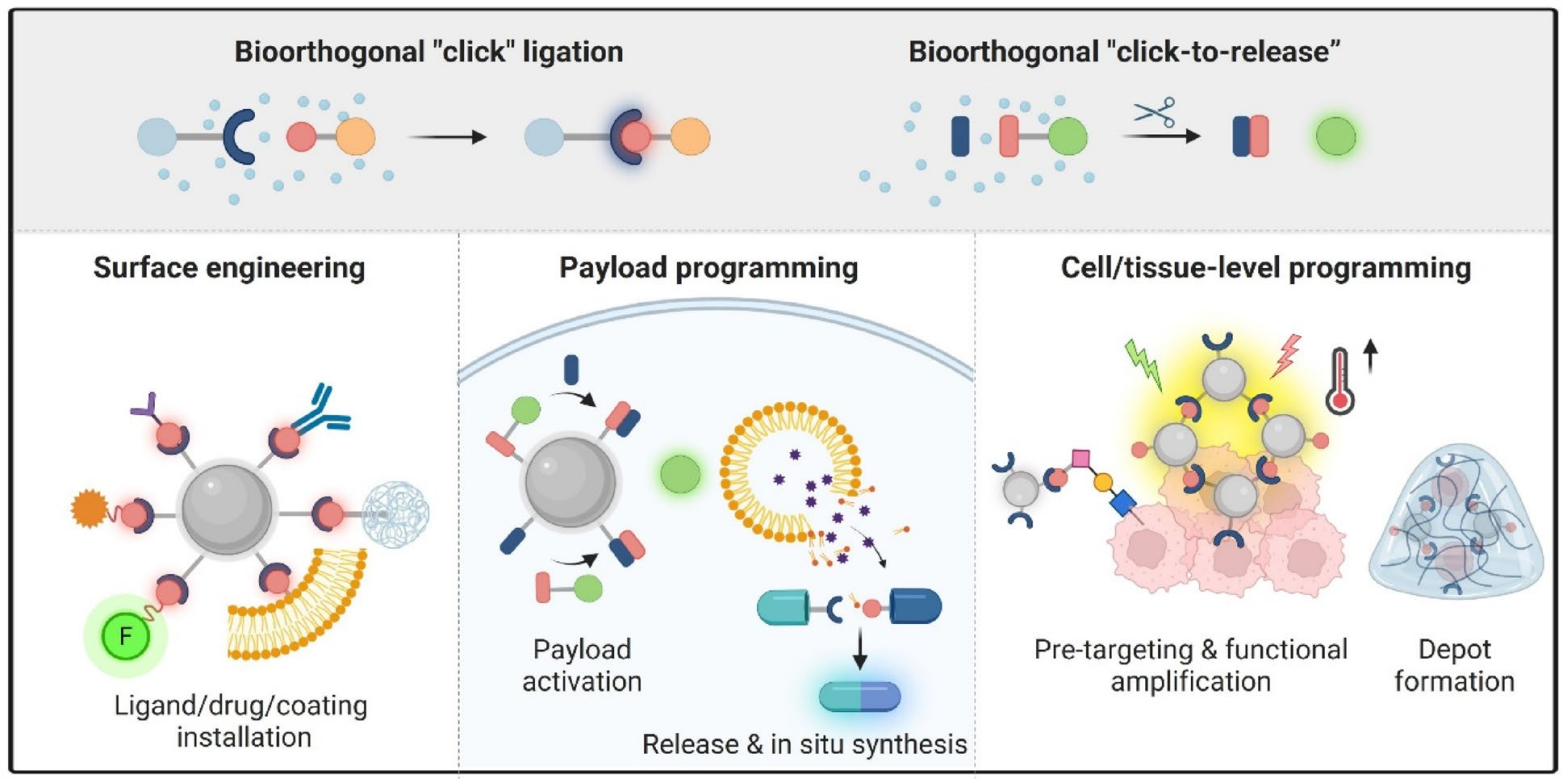

## Introduction

Over the past several decades, nanoparticle (NP)-based biomedical platforms have emerged as powerful tool for the precise diagnosis and treatment of diverse diseases owing to their unique physicochemical properties [1, 2]. Since the approval of pegylated liposomal doxorubicin, Doxil® in 1995 as the first FDA-approved nanomedicine [3], the clinical translation of nanoscale therapeutics carrying diverse drugs has steadily broadened across multiple material classes and disease settings [4–8]. This progress has been driven largely by the capacity of nanoformulations to overcome key limitations of free drugs, including poor solubility, low plasma stability, unfavorable biodistribution, rapid clearance, and off-target toxicity [9–12]. Moreover, as clinically exemplified by Vyxeos®, a liposomal formulation of daunorubicin and cytarabine at a fixed molar ratio [13], nanocarriers can coordinate the delivery of multiple therapeutic agents at predefined ratios, enabling combination strategies that are difficult to achieve with conventional formulations alone [14, 15].

Alongside these advances, the field has increasingly evolved beyond simple drug encapsulation toward chemically and biologically sophisticated nanosystems. Surface modification with biomolecules such as antibodies, peptides, carbohydrates, and aptamers has been widely explored to complement passive accumulation through the enhanced permeability and retention (EPR) effect by promoting biological barrier crossing or receptor-specific recognition [16–18]. In addition, stimuli-responsive design principles have been incorporated to program drug release within defined pathological microenvironments. These platforms can be engineered to respond to exogenous inputs, including light, heat, ultrasound, and magnetic fields, as well as endogenous disease-associated cues such as enzymatic activity, acidic pH, hypoxia, oxidative stress, and redox imbalance, thereby supporting more spatially and temporally controlled therapeutic intervention [19–22]. In parallel, NPs endowed with tailored optical, magnetic, or photophysical characteristics have advanced imaging, sensing, and externally triggered treatment modalities, including photodynamic therapy, photothermal therapy, and sonodynamic therapy [4, 23–28]. More broadly, the performance of contemporary nanomedicine is determined not only by the intrinsic properties of the nanoparticles, but also by the precision with which auxiliary modules are integrated to regulate biodistribution, biointerfacial interactions, and disease context-dependent behavior [29–33]. Accordingly, the modular, efficient, and biocompatible installation of such functional elements has become a central consideration in advanced nanomedicine [12, 34].

Within this context, bioorthogonal chemistry has emerged as a particularly compelling strategy for NP engineering. By definition, bioorthogonal reactions can proceed in complex biological environments without disrupting endogenous biomolecules or native biochemical processes, while maintaining high selectivity, favorable conversion efficiency, and reaction kinetics compatible with physiological conditions [35, 36]. From a synthetic perspective, their high chemoselectivity, mild reaction conditions, and compatibility with functional biomolecules make them especially useful for post-synthetic nanomaterial modification [37, 38]. These features can improve conjugation efficiency and help preserve the activity of sensitive biomolecules compared with conventional carbodiimide, succinimidyl ester, or maleimide coupling approaches. Accordingly, bioorthogonal ligation enables the modular installation of targeting ligands, imaging agents, or therapeutic payloads while minimizing damage to nanostructures, cargoes, or biological ligands [39–42]. Beyond post-synthetic surface functionalization, bioorthogonal chemistry can also be exploited in vivo to direct payload activation, nanoscale assembly, or generate localized functions at disease sites [43, 44]. Whereas stimuli-responsive systems typically rely on endogenous pathological cues or external physical inputs, bioorthogonal chemistry introduces exogenously controllable chemical reactions into biological environments through administered chemical triggers or complementary reaction partners. This reaction-based on-demand activation can help coordinate nanomedicine function with pharmacokinetics and disease-site localization, thereby reducing off-target toxicity and improving therapeutic efficacy.

**Fig. 1:**
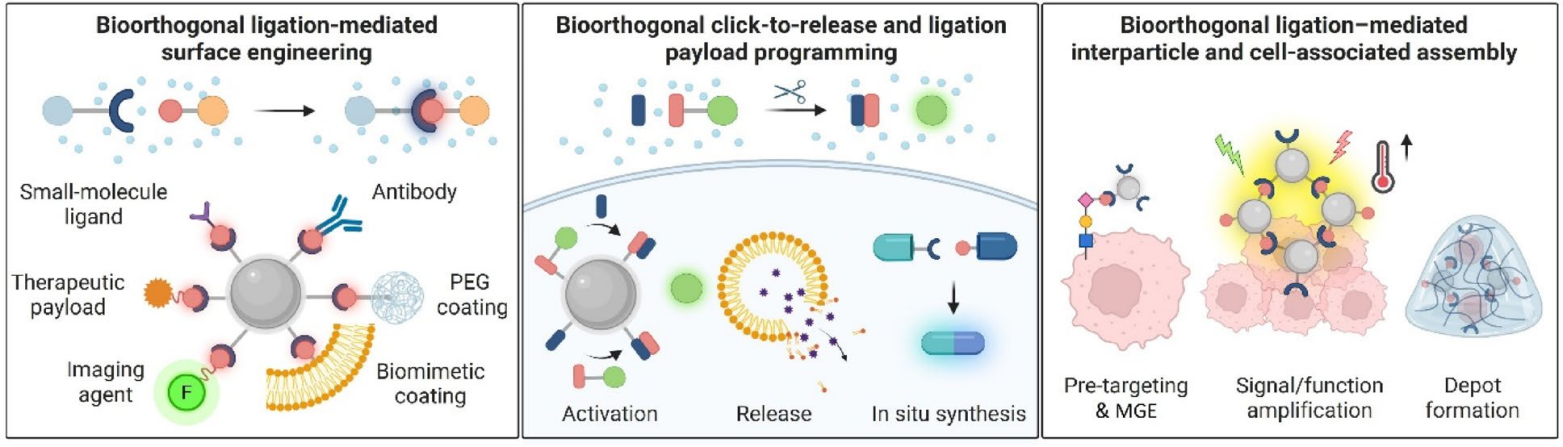
Bioorthogonal chemistry-mediated engineering and in situ modulation of nanoparticle functions

Although bioorthogonal reactions have been extensively reviewed from the perspectives of reaction development and chemical biology, integrated discussions of how these reactions are implemented in nanomedicine remain relatively limited. Here, we adopt a multiscale engineering framework that emphasizes where each reaction operates within the nanomedicine architecture (Fig. 1). At the surface level, bioorthogonal ligation provides modular and mild post-fabrication access to targeting, imaging, and biomimetic interfaces. At the molecular level, click-to-release and bond-forming reactions regulate payload activity with temporal and spatial precision. At the interparticle and cell-associated level, complementary reactive handles can create artificial recognition, localized anchoring, aggregation, depot formation, or immune cell–tumor cell assembly. This scale-based perspective is intended to move the discussion beyond cataloguing clickable nanoplatforms and toward extracting design principles that connect reaction kinetics, biological accessibility, and therapeutic function.

## Types of bioorthogonal reaction

In 2003, Bertozzi and co-workers coined the term bioorthogonal chemistry to describe the conceptual basis of the biocompatible Staudinger ligation developed in the 1990s [45]. Bioorthogonal chemistry encompasses reactions between non-native functional groups that are absent from biological systems, inert to endogenous biomolecules and biochemical processes, and selectively reactive with each other under physiological conditions [46]. Like click chemistry, bioorthogonal reactions are characterized by high selectivity, rapid kinetics, high yields, and mild reaction conditions [47]; however, they are more strictly defined by the requirement for biocompatible reactivity in aqueous physiological environments without cytotoxicity. Owing to these features, bioorthogonal chemistry has become a key tool for precisely labeling and manipulating complex biological systems, thereby greatly expanding the scope of chemical biology. In this chapter, we will focus on bioorthogonal reactions widely used for nanoparticle functionalization and classify them into two categories: (1) ligation reactions, which covalently couple two clickable components, and (2) bioorthogonal click-to-release reactions, which enable selective and controllable bond scission (Table 1).

**Table 1:** Representative bioorthogonal reaction pairs used in nanomedicine

		Reaction pairs	Reaction rates (M^−1^ s^−1^)	Biological compatibility
Bioorthogonal ligation reaction	Staudinger ligation	Azide + phosphine	~ 10^–3^	Chemoselective, but slow and limited by phosphine oxidation
	CuAAC	Azide + alkyne + Cu(I)	10–100	Efficient for ex vivo NP functionalization, but Cu toxicity limits in vivo use
	SPAAC	Azide + cyclooctyne	10⁻^2^– 1	Copper-free and live-system compatible, but cyclooctyne stability/hydrophobicity can affect NP surfaces
	IEDDA	Tetrazine + TCO/dienophile	10^2^ – 10⁶	Fast and in vivo-suitable, but tetrazine degradation and TCO isomerization can limit stability
	Thiol-CBT	CBT + cysteine/aminothiol	1—10	Biocompatible and tumor-triggerable, but needs exposed cysteine
Bioorthogonal click-to-release reaction	Cyclooctene-Tetrazine reaction	Tetrazine + rTCO-payload	1—100	Enables payload release, but release efficiency is structure-dependent
	Vinyl ether-Tetrazine reaction	Tetrazine + vinyl ether-payload	~ 10⁻^4^	Small and stable caging group, but slow; better for local/proximity systems
	Iminosydnones-cyclootyne reaction	Iminosydnone + cyclooctyne	10⁻^2^–10^3^	Metal-free and tunable, but isocyanate byproduct may react with thiols/amines

### Bioorthogonal ligation reaction

#### Staudinger ligation

**Fig. 2:**
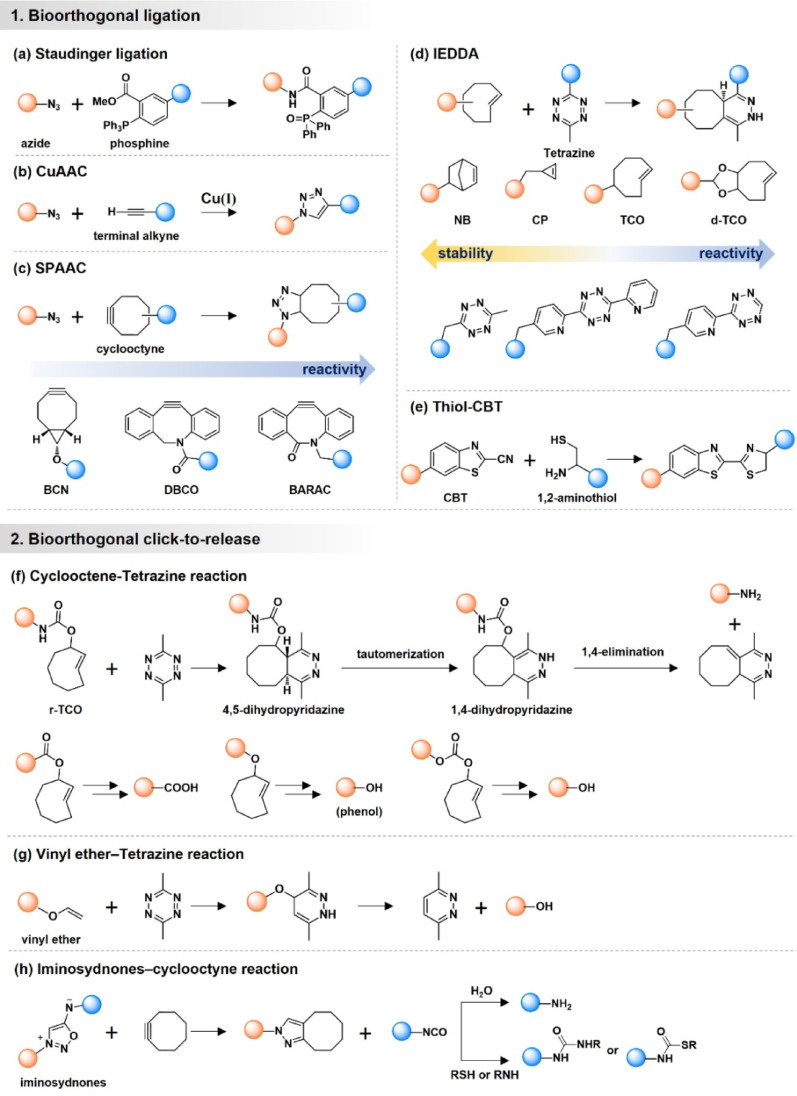
Summary of representative bioorthogonal reactions discussed in this review. BCN:bicyclononyne; DBCO: dibenzocyclooctyne; BARAC: biarylazacyclooctynone; NB: norbornene; CP: cyclopropane; TCO: trans-cyclooctene; d-TCO: dioxolane-fused TCO; CBT: 2-cyanobenzothiazole; rTCO: release-trans-cyclooctene

Staudinger ligation is the earliest bioorthogonal ligation reaction, based on the reaction between azides and phosphine derivatives [48]. By incorporating an electrophilic trap into the classical Staudinger reduction, this chemistry was adapted to form amide linkages between two molecules under physiological conditions (Fig. 2a). Despite its historical importance and excellent chemoselectivity, its practical utility is limited by two major drawbacks. First, phosphines are readily oxidized in biological environments, which compromises reaction efficiency [49, 50]. Second, the reaction is intrinsically slow, typically exhibiting second-order rate constants on the order of ~ 10^–3^ M^−1^ s^−1^ [51], and is therefore not well suited for tracking rapid biological processes in real time. As a result, Staudinger ligation has largely been superseded by faster and more practical cycloaddition-based bioorthogonal reactions and is now less frequently used in bio-applications.

#### CuAAC

Copper(I)-catalyzed azide–alkyne cycloaddition (CuAAC) is a prototypical click reaction in which azides react with terminal alkynes in the presence of Cu(I) to form stable 1,4-disubstituted 1,2,3-triazoles with high regioselectivity (Fig. 2b)[52, 53]. Owing to its fast kinetics, typically in the range of 10–100 M^−1^ s^−1^ [52–54], together with high yields, CuAAC has been widely used for the functionalization of biomolecules and nanomaterials. However, the direct biological application of CuAAC remains constrained by the cytotoxicity of Cu(I), which can induce cellular damage through redox cycling, while byproducts generated from reducing agents such as sodium ascorbate may further contribute to oxidative stress [55, 56]. Consequently, although CuAAC is highly effective for ex vivo modification and post-synthetic nanomaterial functionalization, its direct in vivo use remains limited. More recently, bioorthogonal nanozymes, in which catalytic metals are confined within nanoparticle scaffolds, have emerged as a promising strategy to improve the biological applicability of CuAAC [57].

#### SPAAC

Strain-promoted azide–alkyne cycloaddition (SPAAC), introduced by Bertozzi and co-workers in 2004, is a copper-free cycloaddition in which a strained cyclooctyne reacts fastly with an azide without the need for a metal catalyst (Fig. 2c) [58]. Because SPAAC proceeds efficiently under physiological conditions and avoids catalyst-associated toxicity, it has become a representative bioorthogonal ligation reaction applicable to living systems, including live cells [50, 58], zebrafish [59], and mice [60]. Early SPAAC systems displayed relatively slow kinetics, with rate constants of approximately ~ 10^–2^ M^−1^ s^−1^ [61]. However, the subsequent development of more reactive cyclooctyne derivatives, including bicyclononyne (BCN), dibenzocyclooctyne (DBCO), and biarylazacyclooctynone (BARAC), substantially improved reaction performance. For example, the DBCO–azide pair typically exhibits second-order rate constants of 0.2–0.5 M^−1^ s^−1^, whereas BARAC reaches approximately 0.96 M^−1^ s^−1^ [61]. Nevertheless, enhancement of cyclooctyne reactivity is often accompanied by reduced stability, increased hydrophobicity, and greater synthetic complexity. These trade-offs become particularly important in nanoparticle surface engineering, where colloidal stability and surface accessibility can be directly affected by the properties of the strained alkyne. Accordingly, the design of SPAAC-based nanoplatforms should balance reactivity with aqueous solubility, effective surface presentation, and nanoparticle stability.

#### IEDDA

The inverse electron-demand Diels–Alder (IEDDA) reaction, also known as tetrazine ligation, proceeds through cycloaddition between an electron-deficient tetrazine and a strained dienophile, followed by a retro-Diels–Alder process accompanied by N_2_ extrusion (Fig. 2d) [62]. Since its introduction in 2008, this reaction has become a central platform in bioorthogonal chemistry. The most widely used reaction pair is tetrazine and *trans*-cyclooctene (TCO)[63–65], although norbornene [66], cyclopropene [67, 68], terminal alkene [69] derivatives can also serve as dienophiles. IEDDA is regarded as the fastest bioorthogonal ligation reported to date, with rate constants reaching up to 10^6^ M^−1^ s^−1^ depending on the reactant structures and conditions [70]. Such ultrafast kinetics allow efficient conjugation even at low concentrations and short residence times, making this chemistry particularly advantageous for live-cell labeling, pretargeting, molecular imaging, and in vivo nanomedicine. However, high reactivity is often accompanied by limited stability; highly reactive tetrazines are generally more susceptible to degradation in aqueous media and in the presence of thiols. For example, dimethyltetrazine undergoes approximately 50% hydrolysis within 14 h in PBS, and dipyridyl-tetrazine exhibits a half-life of about 9 h [71], whereas selected alkyl- or pyridinyl-substituted tetrazines show substantially improved stability [72]. Likewise, TCO, the most commonly used dienophile, can isomerize to the unreactive cis-form in the presence of thiols or serum proteins, thereby compromising its reactivity during circulation [73, 74]. To address this limitation, more stable dienophile structures, such as dioxolane-fused TCO derivatives, have been developed [75]. Therefore, successful implementation of IEDDA in nanoparticle functionalization requires careful consideration not only of reaction kinetics, but also of the aqueous solubility and serum stability of both reaction partners, their mode of surface presentation, and the colloidal stability of the resulting nanoconjugates.

#### Thiol-CBT reaction

The thiol-CBT reaction is a condensation reaction between 2-cyanobenzothiazole (CBT) and a 1,2-aminothiol, most commonly a cysteine derivative. Mechanistically, the reaction is initiated by nucleophilic attack of the thiol on the cyano group, followed by intramolecular cyclization to generate a thiazoline-type product (Fig. 2e). This chemistry was first applied by Rao et al. to protein labeling and was shown to proceed with relatively rapid kinetics, with a second-order rate constant of approximately 9.19 M^−1^ s^−1^ [76]. In addition, cysteine derivatives can be readily incorporated via protecting-group strategies, enabling the design of conditional bioorthogonal systems activated by tumor-associated stimuli, such as enzymes, pH, and glutathione (GSH) [76]. Owing to these features, the thiol-CBT reaction has attracted considerable attention as a highly biocompatible condensation-based ligation chemistry useful for tumor-selective self-assembly and signal amplification.

### Bioorthogonal click-to-release reaction

#### Cyclooctene-TETRAZINE reaction

The cyclooctene-tetrazine click-to-release reaction is a representative bioorthogonal bond-cleavage strategy based on the IEDDA reaction between tetrazine and release-*trans*-cyclooctene (rTCO) bearing a payload at the allylic position (Fig. 2f). First reported by Robillard and co-workers in 2013, this reaction proceeds through formation of a 4,5-dihydropyridazine intermediate, which subsequently tautomerizes to the 1,4-dihydropyridazine form; the latter then undergoes 1,4-elimination to release the payload [71]. By incorporating appropriate self-immolative linkers, this strategy has been extended to the release of cargos containing secondary amines [77, 78], carboxylic acids [79], phenols [80, 81], and alcohols [82]. The reaction kinetics and overall release efficiency vary widely depending on the structures of both tetrazine and TCO [83]. Importantly, rapid ligation does not necessarily result in efficient payload release, as highly reactive tetrazines may also form non-releasing dead-end isomers. Subsequent structure–activity studies showed that unsymmetrical tetrazines bearing an electron-withdrawing substituent together with a small alkyl group can provide a more favorable balance between fast ligation and high release yield [84].

#### Vinyl ether–Tetrazine reaction

An IEDDA reaction between tetrazine and a vinyl ether can trigger the release of alcohol-, phenol- or amine-containing payloads protected as vinyl ethers (Fig. 2g) [85–87]. Following the relatively slow initial cycloaddition, the reaction proceeds through subsequent fragmentation steps to release the final payload. Compared with tetrazine-triggered cleavage of TCO derivatives, this system is kinetically slower, with representative second-order rate constants of approximately 10^–4^ M^−1^ s^−1^ [87]. Nevertheless, vinyl ethers offer several advantages, including small structural size, synthetic accessibility, and relatively good stability as protecting groups. Consequently, although the vinyl ether–tetrazine system is less suitable for systemic in vivo cleavage, it may be advantageous in proximity-driven systems or nanostructured theranostic platforms, where local enrichment of the reaction partners can compensate for the inherently slower kinetics.

#### Iminosydnones–cyclooctyne reaction

The iminosydnone–cyclooctyne reaction is an emerging click-to-release platform based on strain-promoted iminosydnone–cycloalkyne cycloaddition (SPSIC), in which a mesoionic iminosydnone reacts with a strained cyclooctyne through a [3 + 2] cycloaddition to trigger payload release (Fig. 2h) [88]. Mechanistically, the initially formed cycloadduct undergoes rearrangement to generate a pyrazole while releasing an isocyanate intermediate, which is subsequently hydrolyzed to furnish the final amine-containing product. Notably, the liberated isocyanate may react not only to water but also with endogenous thiols or amines in biological environments, thereby forming thiocarbamate or urea derivatives. The reaction kinetics are highly dependent on the cargo structure, cyclooctyne architecture, solvent composition, and pH, with reported rate constants generally spanning 10^–2^–10^3^ M^−1^ s^−1^ [89–92]. Thus, this chemistry can achieve relatively fast click kinetics through structural optimization, but its overall performance must be considered in light of both the reactivity–stability trade-off and the downstream reactivity of the liberated isocyanate. Although iminosydnone–cyclooctyne chemistry is not yet as broadly established as the tetrazine–TCO platform, its metal-free nature and design flexibility make it a promising candidate for next-generation click-to-release systems.

Taken together, the effective performance of a bioorthogonal pair for NP functionalization is determined by the combined effects of intrinsic kinetics, aqueous and serum stability, steric accessibility at the nanointerface, hydrophobicity of the clickable handle, and compatibility with the intended biological route. For example, IEDDA chemistry is attractive for low-concentration in vivo ligation, but tetrazine degradation and TCO isomerization can limit the amount of reactive material available during circulation. By contrast, SPAAC avoids the need for added catalysts and benefits from relatively stable azide handles, but its slower kinetics can limit in vivo applications requiring efficient ligation across diverse biological time scales; bulky hydrophobic cyclooctynes may also perturb nanoparticle colloidal stability or surface presentation. Click-to-release systems impose an additional requirement: rapid ligation must be coupled to efficient productive elimination rather than formation of non-releasing adducts. Therefore, reaction choice in programmable nanomedicine should be matched to the local concentration regime, residence time, and desired output, rather than treated as an interchangeable labeling step.

## Bioorthogonal surface engineering across nanomaterial platforms

**Fig. 3:**
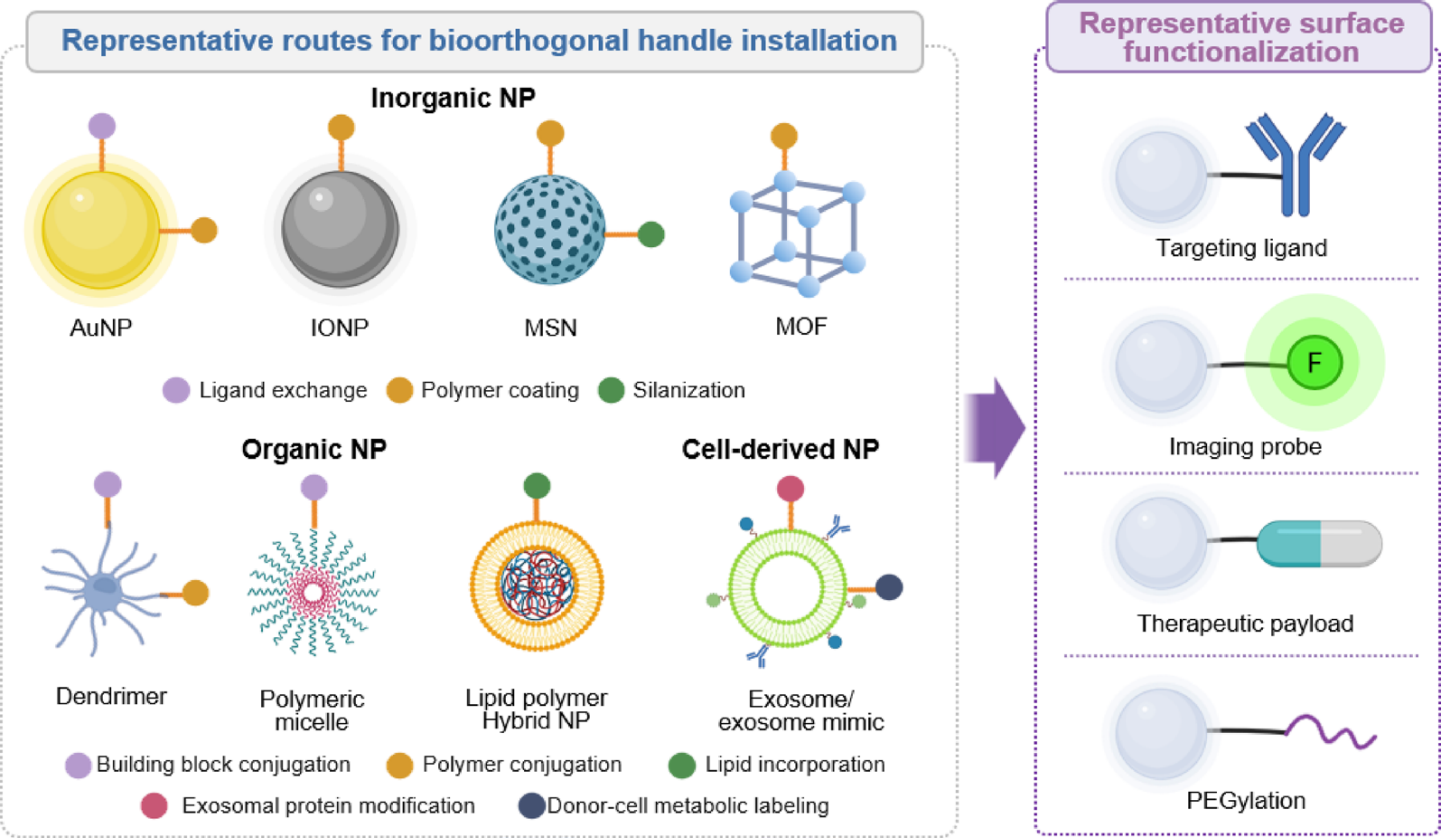
Representative strategies for bioorthogonal surface engineering of nanomedicine platforms

Bioorthogonal ligation provides a straightforward strategy for introducing diverse functional moieties, including fluorophores, contrast agents, targeting ligands, and cytotoxic drugs, without compromising carrier integrity or ligand functionality (Fig. 3). In this chapter, we highlight representative examples of nanomaterial surface functionalization, with particular emphasis on how bioorthogonal handles were incorporated into each nanomaterial during NP synthesis or post-synthetic modification and the specific purpose they serve (Table 2).

**Table 2:** Representative bioorthogonal surface engineering strategies across nanomaterial platforms

	Platform	Reaction type	Reaction condition	Installed module	Refs
AuNPs	Thiol–triphenylphosphine-modified AuNPs	Staudinger ligation	CDCl₃, 25 °C	CRGDK peptide	[103]
	Alkene-bearing Au@HMBPene NPs	IEDDA	Aqueous condition, RT, overnight	Sulfo-Cy5	[104]
	HAuNP–DNBA–N₃	SPAAC	PBS buffer/DMSO, RT, 1 h	BCN–folate	[105]
	Azido-PEG-NHC@AuNPs	SPAAC	H₂O/EtOH, 3h	FITC	[106]
	GNR–PEG–DBCO	SPAAC	Aqueous condition, 20 °C, 4 h	Anti-DR4 antibody	[107]
	BCN-functionalized AuNPs	SPAAC	Aqueous condition, RT, 2 h	OMV / anti-EGFR scFv	[108]
IONPs	Tz-modified CLIO NPs	IEDDA	Aqueous condition, RT, 3 h	trastuzumab / cetuximab / anti-EpCAM	[114]
	Azide-SPION nanoemulsion	SPAAC	PBS buffer, RT, 12 h	Anti-HER2 affibody	[116]
	DBCO-/cyclooctyne-IONPs	SPAAC	Aqueous or cell culture media condition, 37 °C, 1–6 h	PEG, coumarin	[117]
	DBCO-MNPs	SPAAC	PBS buffer, RT, 12 h	EGFR-rich cell membrane	[120]
MSNs	Norbornene-CA-capped MSNs	IEDDA	HBSS buffer, RT, 2 h	Folic acid	[123]
	Azide-MSNs	IEDDA	PBS buffer, RT, 1 h	CPD / ASO / drug	[124]
	Cyclopropene/maleimide SNPs	IEDDA	CH₃CN, RT	Radioiodine / Cy5.5	[125]
MOFs	DBCO-DPMA-coated NMOFs	SPAAC	Aqueous condition, 37 °C, 30 min	PEG / mannose	[132]
Dendrimers	Azide-terminal poly(amido) dendrimers	SPAAC	CH₂Cl₂, RT	PEG	[135]
	Glucose-rich dendrimer scaffold	IEDDA	DMF, RT, 2 h	Glucose units	[136]
	Tz-capped PEG polylysine dendrimer	IEDDA	PBS buffer, 37 °C, overnight	Anti-HER2 nanobody	[137]
	Azide-bearing AmD micelles	SPAAC	Aqueous condition, RT, 4 h	MEO-PEG-CDM-DBCO / DOX	[138]
Micelles and hybrid NPs	Azide-PEG-PLGA NPs	SPAAC	PBS buffer, 37 °C, 30 min	Antibodies (Anti-EGFR / Anti-CD16 / Anti -4-1BB)	[143]
	Azide- or Tz-functionalized nanogels	SPAAC / IEDDA	Aqueous condition, RT, 2–72 h	Trastuzumab	[144]
	Maleimide/Tz/N₃-lipid hybrid NPs	Thiol–maleimide / IEDDA / SPAAC	PBS buffer, 0.5–2 h	OVA / CRM197 / sKLH	[145]
Cell-derived exosomes and nanovesicles	Alkyne-modified 4T1 exosomes	CuAAC	PBS buffer, RT, 3 h	Fluorophore	[156]
	DBCO-modified MSC exosomes	SPAAC	PBS buffer, 4 °C, 12 h	c(RGDyK) peptide	[157]
	Azide-choline-labeled M1 Exo-N₃	SPAAC	Exosome-free medium, 48 h	Anti-CD47 antibody / anti-SIRPα antibody	[158]
	DS-EXOs from metabolically engineered ADSCs	SPAAC	Serum-free medium, 2 h	Dextran sulfate	[159]
	N₃-displayed MSC exosome mimetics	SPAAC	PBS buffer, RT, 15 min	Alendronate	[160]
	Azido-tagged EVs	SPAAC	PBS buffer, 4 °C, 4 h	Cy3 / Cy5 / CpG	[161]
	N₃-Ino-labeled exosomes	SPAAC	RT, 1 h	Fluorophores	[162]

### Inorganic nanomaterials

**Fig. 4:**
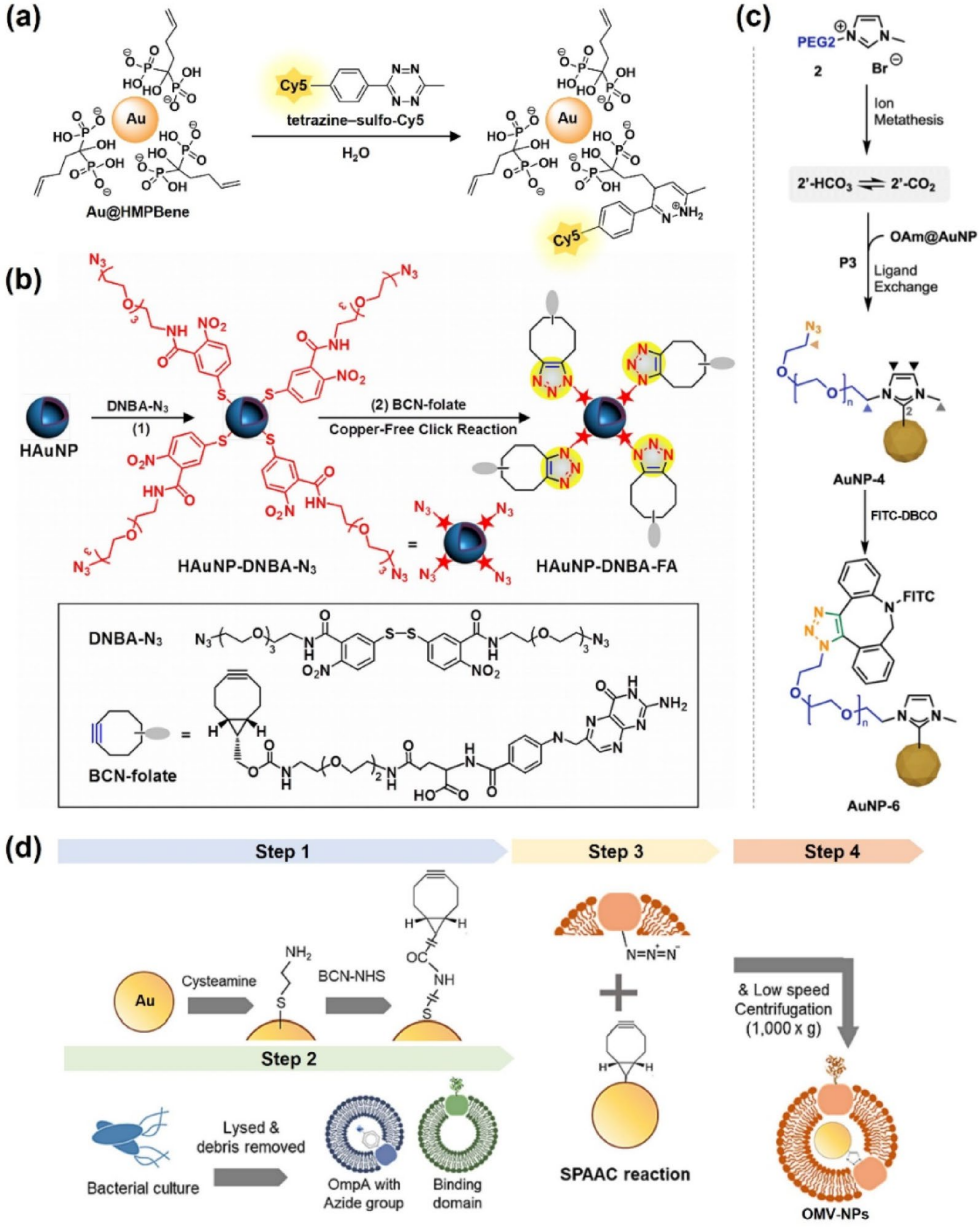
**a** Schematic illustration of HMPBene-gold NP surface modification via the IEDDA reaction. Adapted from Ref. [104] with permission from Wiley–VCH, copyright 2016. **b** Schematic illustration of SPAAC-mediated synthesis of HAuNP–DNBA–FA. Reprinted from Ref. [105] with permission from the American Chemical Society, copyright 2017. **c** Schematic illustration of FITC conjugation to the AuNP surface via SPAAC using an NHC ligand. Adapted from ref. [106] with permission from The Royal Society of Chemistry, copyright 2024. **d** Schematic illustration of SPAAC-mediated synthesis of OMV-NPs. Reprinted from Ref. [108] with permission from Elsevier Inc, copyright 2024

Following the early 1970s introduction by Faulk and Taylor of intracellular molecular visualization using gold nanoparticle–antibody conjugates, research into the biomedical applications of metal-based NPs has expanded substantially [93]. In particular, gold nanoparticles (AuNPs) have become central nanomaterials in biomedicine because of their high biocompatibility and distinctive plasmonic properties, enabling applications in bioimaging, biosensing, photothermal therapy, and photodynamic therapy [94–102]. In 2015, Workentin et al. provided an early example of AuNP functionalization using bioorthogonal chemistry, where thiol-linked triphenylphosphine was introduced onto AuNPs via thiol–gold affinity, followed by conjugation with an azide-linked 5-mer peptide via Staudinger ligation [103]. Guenin et al. reported AuNP surface functionalization with a near-infrared (NIR) fluorophore via IEDDA chemistry [104]. In this approach, alkene handles were incorporated during AuNP synthesis to generate Au@HMBPene using a bisphosphonate linker, exploiting its metal-chelating properties; the pendant alkene was subsequently conjugated with tetrazine–sulfo-Cy5 through the IEDDA reaction (Fig. 4a). This study presented a straightforward strategy to install both bioorthogonal handles and imaging probes on the AuNP surface. Zhang et al. prepared folate-targeted surface-enhanced Raman scattering (SERS) nanoprobes from hollow gold nanoparticles (HAuNPs) using SPAAC chemistry [105]. In this design, the gold surface was first modified with DNBA-N_3_, a Raman-active disulfide bearing a terminal azide (Fig. 4b). The disulfide anchored the Raman label to the gold surface, whereas the azide served as the bioorthogonal handle for subsequent conjugation with BCN–folate via SPAAC. The resulting HAuNP–DNBA–FA probes exhibited strong SERS signals and selectively recognized folate receptor-positive cancer cells. A more robust post-synthetic platform was later reported by Reithofer et al., who investigated highly stable N-heterocyclic carbene (NHC) ligands to introduce terminal azides onto AuNPs [106]. Their strategy started from well-defined oleylamine-protected AuNPs (OAm@AuNP), which underwent top-down ligand exchange with azide-labeled NHC precursors (Fig. 4c). This process yielded a SPAAC-ready AuNP scaffold (AuNP-4) for subsequent conjugation with FITC-labeled DBCO for biomedical applications. Micheau et al. reported stepwise post-synthetic modification of gold nanorods (GNRs) with an anti-DR4 antibody via SPAAC [107]. GNRs were first conjugated with HS-PEG10K-COOH through gold–sulfur affinity, followed by amide coupling with DBCO-NH_2_ to generate GNR-PEG10K-DBCO. The resulting nanorods were then conjugated with azide-bearing DR4 antibodies via SPAAC. Notably, this work demonstrated that SPAAC better preserved antibody bioactivity than conventional conjugation approaches, including amide coupling and Schiff-base chemistry. A distinct form of gold-surface engineering was reported by Jose et al., who used bioorthogonal chemistry to couple outer membrane vesicles (OMVs) to AuNPs (Fig. 4d) [108]. In this approach, AuNPs were functionalized sequentially with cysteamine and BCN-succinimidyl ester (BCN-NHS), thereby introducing a strained alkyne onto the particle surface. In parallel, the complementary azide functionality was installed biologically by incorporating p-azidophenylalanine into membrane proteins (OmpA) within the OMV membrane. Subsequent SPAAC yielded covalently linked OMV–AuNP hybrids. The resulting OMV-NPs retained membrane-associated functions, such as Nox activity, and could also display anti-EGFR scFv for tumor-cell targeting.

**Fig. 5:**
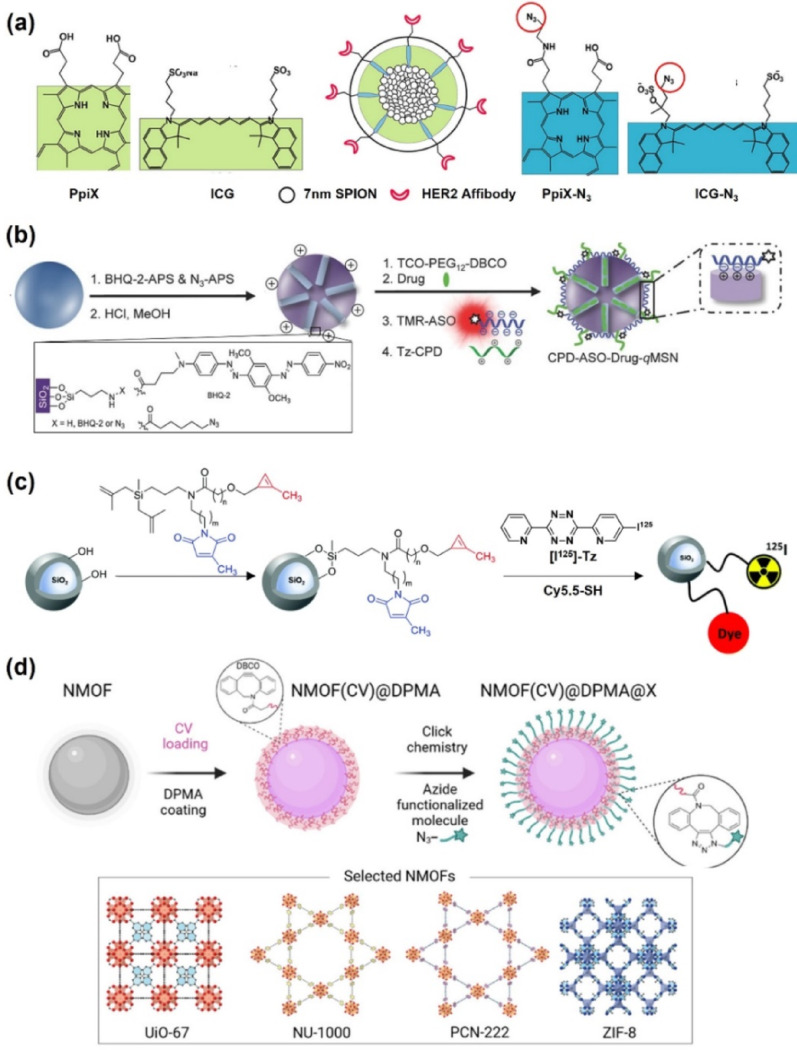
**a** Schematic illustration of an SPION nanoemulsion coated with azide-labeled amphiphilic dyes. Adapted from Ref. [116] with permission from the American Chemical Society, copyright 2018. **b** Schematic illustration of the sequential SPAAC- and IEDDA-mediated synthesis of CPD-ASO-drug-qMSN. Reprinted from Ref. [124] with permission from Wiley–VCH, copyright 2017. **c** Schematic illustration of the one-pot synthesis of dual-functional SNPs. Adapted from ref. [125] with permission from The Royal Society of Chemistry, copyright 2022. **d** Schematic illustration of the SPAAC-mediated surface modification of selected NMOFs. Reprinted from Ref. [132] with permission from the American Chemical Society, copyright 2025

Owing to their magnetic responsiveness and favorable biocompatibility, iron oxide nanoparticles (IONPs) have been widely used in biomedicine, particularly for bioimaging, drug delivery, and theranostic applications [109–113]. In an early study, Weissleder et al. reported dextran-coated crosslinked iron oxide (CLIO) NPs bearing primary amine groups, which were further modified with NHS-tetrazine via amide coupling [114]. Using IEDDA chemistry, TCO-labeled antibodies against HER2, EpCAM, and EGFR were conjugated to the CLIO surface, enabling efficient targeting of cancer cells expressing the corresponding receptors. A similar approach was later reported by He et al., who developed tetrazine-functionalized IONPs via amide coupling between a carboxylic acid–functionalized IONP precursor (prepared by oxidation of the oleic acid ligand) and a tetrazine-linked amine [115]. This strategy introduced bioorthogonal handles onto the IONP surface through a simple, reproducible, and scalable route. Tsourkas et al. produced azide-modified superparamagnetic IONP (SPION) nanoemulsions by simply mixing hydrophobic SPIONs with azide-bearing amphiphilic dyes (Fig. 5a) [116]. The azide handles, linked to a hydrophilic linker, were exposed on the surface and subsequently conjugated to a DBCO-labeled anti-HER2 affibody through SPAAC, yielding a cancer-targeting nanoemulsion. This system selectively bound HER2-positive cancer cells and generated clear T2-weighted MR contrast. Similarly, Fratila et al. reported a two-step post modification strategy [117]. In this work, IONPs were initially coated with amphiphilic poly(maleic anhydride-alt-1-octadecene) (PMAO), following a previously reported procedure [118, 119]. The surface-exposed hydrophilic maleic anhydride groups were subsequently functionalized, via carbodiimide coupling, with either cyclooctynylamine or DBCO-NH₂ as bioorthogonal handles and with glucopyranoside (GLC) or PEG as passivating moieties. A series of solution-phase studies revealed that GLC@DBCO-functionalized particles exhibited superior SPAAC reactivity, compared with cyclooctyne-based analogues, toward various azide-modified substrates, including PEG, a coumarin fluorophore, and azide-modified gold surfaces. More recently, Li et al. reported the use of bioorthogonal ligation to install membrane-derived biological interfaces on magnetic IONPs (MNPs) [120]. In this study, Fe_3_O_4_@SiO_2_–NH_2_ particles were modified with DBCO-NHS to generate DBCO-bearing MNPs, which were subsequently reacted with azide-labeled, EGFR-rich cell membranes prepared through metabolic glycoengineering. The resulting EGFR/MNPs were used as magnetic probes for EGFR-oriented capture of chemical hazards, illustrating the potential of bioorthogonal ligation to endow nanoplatforms with biomimetic properties through covalent membrane coating.

Mesoporous silica nanoparticles (MSNs) have been widely used as nanocarriers for the delivery of therapeutic, diagnostic, and theranostic agents owing to their well-defined and tunable particle size, pore architecture, and morphology, together with their versatile surface chemistry and favorable biocompatibility [121, 122]. In the context of bioorthogonal chemistry, MSNs are particularly attractive because clickable functionalities can be introduced either directly onto the silica surface through organosilane-based modification or indirectly through surface-associated interfacial components. Bein et al. reported a representative example of surface-adjacent bioorthogonal functionalization using a carbonic anhydrase (CA)-capped MSN platform [123]. In this design, aryl sulfonamide-functionalized MSNs were used to reversibly anchor CA, while the bioorthogonal handle was introduced onto the CA cap through genetic incorporation of a norbornene-containing unnatural amino acid rather than onto the silica surface itself. Subsequent IEDDA ligation with a tetrazine-modified folic acid ligand enabled installation of a cancer-targeting functionality while preserving the pH-responsive gating behavior of CA. A more direct surface-functionalization approach was reported by Yao et al., who used MSNs as a nanoquencher platform for theranostic intracellular mRNA sensing and drug release [124]. In this system, azide groups were introduced directly onto the MSN surface by incorporating azide-modified 3-aminopropyltriethoxysilane (N_3_-APS), followed by SPAAC with TCO-PEG_12_-DBCO to install cell-penetrating poly(disulfide)s (CPDs) via orthogonal tetrazine (Tz)–TCO ligation with Tz-labeled CPD (Tz-CPD) (Fig. 5b). The resulting CPD-modified MSNs underwent rapid cellular internalization via an endocytosis-independent pathway, thereby enabling intracellular mRNA detection in conjunction with the release of antisense oligonucleotides (ASOs) and therapeutic agents. Min et al. developed a bifunctional silica nanoparticles (SNPs) based on a methallylsilane linker bearing cyclopropene and methylmaleimide groups (Fig. 5c) [125]. By exploiting tetrazine cycloaddition and thiol-conjugate addition, the authors introduced both a radioiodinated imaging module and a Cy5.5 fluorophore, generating a dual-functional SNPs for fluorescence and nuclear imaging in vivo. Notably, this work demonstrated that bioorthogonal-compatible handles can be incorporated into a stable allylsilane precursor and transferred to silica as a one-pot bifunctional platform.

Metal–organic frameworks (MOFs) are porous crystalline materials that form three-dimensional networks with highly ordered and tunable internal structures [126–128]. Owing to their large porosity and high loading capacity for diverse biomolecules, MOFs have emerged as promising nanomedicine platforms for drug delivery [129–131]. To enable facile post-synthetic surface engineering, Pino et al. reported a universal clickable coating strategy applicable to various nanosized MOFs (NMOFs), including UiO-67, NU-1000, PCN-222, and ZIF-8 (Fig. 5d) [132]. In this approach, a DBCO-bearing amphiphilic poly(isobutylene-alt-maleic anhydride)-g-dodecyl (DPMA) polymer was applied as an external coating layer and subsequently conjugated with azide-functionalized surface modules (e.g., PEG, mannose, and a dynein-binding cell-penetrating peptide) via SPAAC chemistry. This study is noteworthy because it demonstrates that bioorthogonal chemistry can transform framework nanocrystals into multifunctional platforms via facile post-synthetic modification.

### Organic nanomaterials

**Fig. 6:**
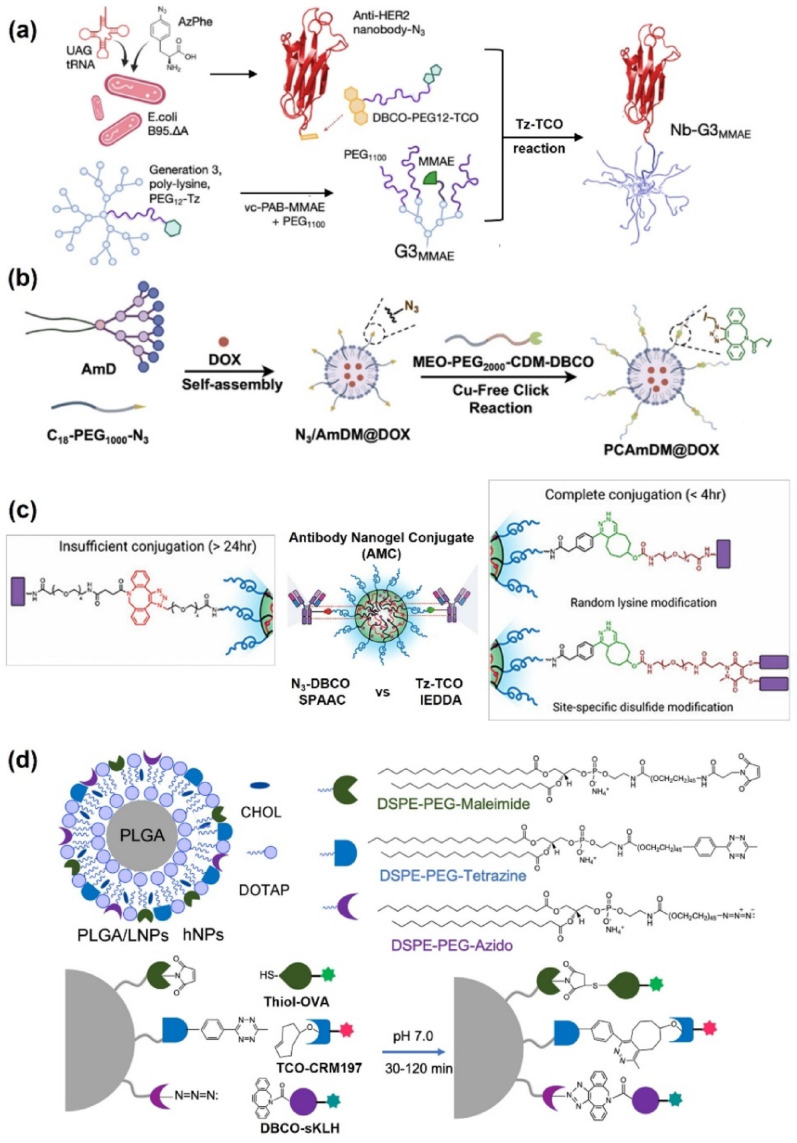
**a** Schematic illustration of single nanobody-conjugated dendrimer synthesis. Reprinted from Ref. [137] with permission from the American Chemical Society, copyright 2025. **b** Schematic illustration of the SPAAC-mediated functionalization of a dendrimer with a pH-responsive PEG module. Reprinted from Ref. [138] with permission from Elsevier Inc, copyright 2026. **c** Comparison of SPAAC- and IEDDA-mediated antibody labeling on the nanogel surface. Reprinted from Ref. [144] with permission from the American Chemical Society, copyright 2023. **d** Structure of PLGA/LNPs and schematic illustration of the sequential conjugation of three different proteins onto a single hybrid NP. Adapted from Ref. [145] with permission from Wiley–VCH, copyright 2025

Owing to their precisely defined architecture, low polydispersity, and multivalent surface functionality, dendrimers have attracted considerable interest in nanomedicine, particularly for targeted drug delivery [133]. However, broader biomedical application has often been limited by synthetic complexity and by the difficulty of introducing targeting or adaptive surface functionalities in a clean and structurally well-defined manner [134]. In 2010, Weck et al. reported the first example of using SPAAC for dendrimer functionalization: they installed multiple azide groups at the terminal sites of poly(amido)-based dendrons/dendrimers and showed that SPAAC enables mild, metal-free, high-yield conjugation with cyclooctyne-functionalized PEG [135]. More recently, Sharma et al. employed sequential thiol–ene, SPAAC, and IEDDA reactions to construct a highly functionalized glucose-rich dendrimer, using bioorthogonal chemistry both to assemble the dendritic framework and to install 60 peripheral glucose units for GLUT-mediated, cancer-targeted uptake [136]. Bioorthogonal chemistry has also enabled precise installation of targeting proteins on dendrimer-based drug carriers. Johnston et al. reported PEGylated polylysine dendrimers carrying the anticancer drug monomethyl auristatin E (MMAE) together with a single anti-HER2 nanobody (Nb-G3_MMAE_) (Fig. 6a) [137]. In this system, the dendrimer core contained a tetrazine-capped PEG linker that served as a defined attachment site, whereas the nanobody was engineered to carry a site-specific azidophenylalanine residue at its C-terminus. The nanobody was first conjugated to a DBCO-PEG_12_-TCO linker via SPAAC and then ligated to the tetrazine-bearing dendrimer. This strategy enabled the programmed installation of one nanobody per dendrimer in a defined orientation, thereby facilitating uptake by HER2-positive cancer cells and subsequent intracellular delivery of MMAE. Liu et al. introduced azide functional groups onto dendrimers by self-assembling an azide-bearing amphiphilic polymer (C_18_-PEG_1000_-N_3_) with a poly(amidoamine) dendrimer (AmD) core (Fig. 6b) [138]. The surface-exposed azide groups were subsequently used to install an acid-responsive PEG linker (MEO-PEG_2000_-CDM-DBCO) via SPAAC, generating a pH-removable PEG shell. In this case, bioorthogonal chemistry imparted an adaptive surface feature that stabilized the dendrimer micelles during blood circulation but detached in the acidic tumor microenvironment. Functionally, this design enabled a size-transformable surface program, illustrating that bioorthogonal chemistry can be applied to dynamically regulate nanocarrier surface behavior.

Micelles and lipid/polymer hybrid NPs are another class of organic NPs that has been widely investigated in nanomedicine because of their core–corona structure with high drug loading capacity and readily tunable properties, including size, shape, degradation, and stimulus responsiveness. These properties make them versatile carriers for efficient and controlled drug delivery to diseased tissues [139–142]. In an early study, Wang et al. prepared azide-functionalized poly(ethylene glycol)–block-poly(lactide-co-glycolide) (PEG-PLGA) NPs and subsequently conjugated them with DBCO-linked anti-EGFR antibodies to target EGFR-positive cancer cells, as well as with anti-CD16 and anti-4-1BB antibodies to activate natural killer (NK) cells [143]. Thayumanavan et al. compared the reaction efficiencies of SPAAC and IEDDA for antibody conjugation to polymer nanogels (Fig. 6c) [144]. Azide or tetrazine bioorthogonal handles were introduced onto a common amine-terminated RAFT polymer precursor by reaction with azide-PEG_4_-NHS or tetrazine-NHS, respectively. In parallel, DBCO- or TCO-modified trastuzumab (a HER2-targeting antibody) was prepared. The authors showed that the TCO-tetrazine IEDDA reaction outperformed the azide-DBCO SPAAC pair, enabling faster functionalization of HER2-targeted nanogels, simplified purification, and improved cellular targeting. A further example was provided by Zhang et al., who demonstrated the facile installation of three distinct proteins onto PLGA/lipid hybrid NPs using orthogonal bioorthogonal reactions (Fig. 6d) [145]. These particles consisted of a PLGA core and a lipid shell incorporating different reactive lipids, including maleimide, tetrazine, and azide, which enabled stepwise surface conjugation of thiol-bearing ovalbumin (OVA), TCO-modified cross-reactive material 197 (CRM197), and DBCO-modified subunit keyhole limpet hemocyanin (sKLH). By combining maleimide–thiol Michael addition, tetrazine–TCO ligation, and azide–DBCO click chemistry, the authors demonstrated selective and sequential attachment of three fluorescently labeled proteins onto a single hybrid NP. This study highlights how bioorthogonal chemistry can expand the surface protein-display capacity of organic NPs beyond conventional monovalent functionalization.

### Cell-derived exosomes and nanovesicles

**Fig. 7:**
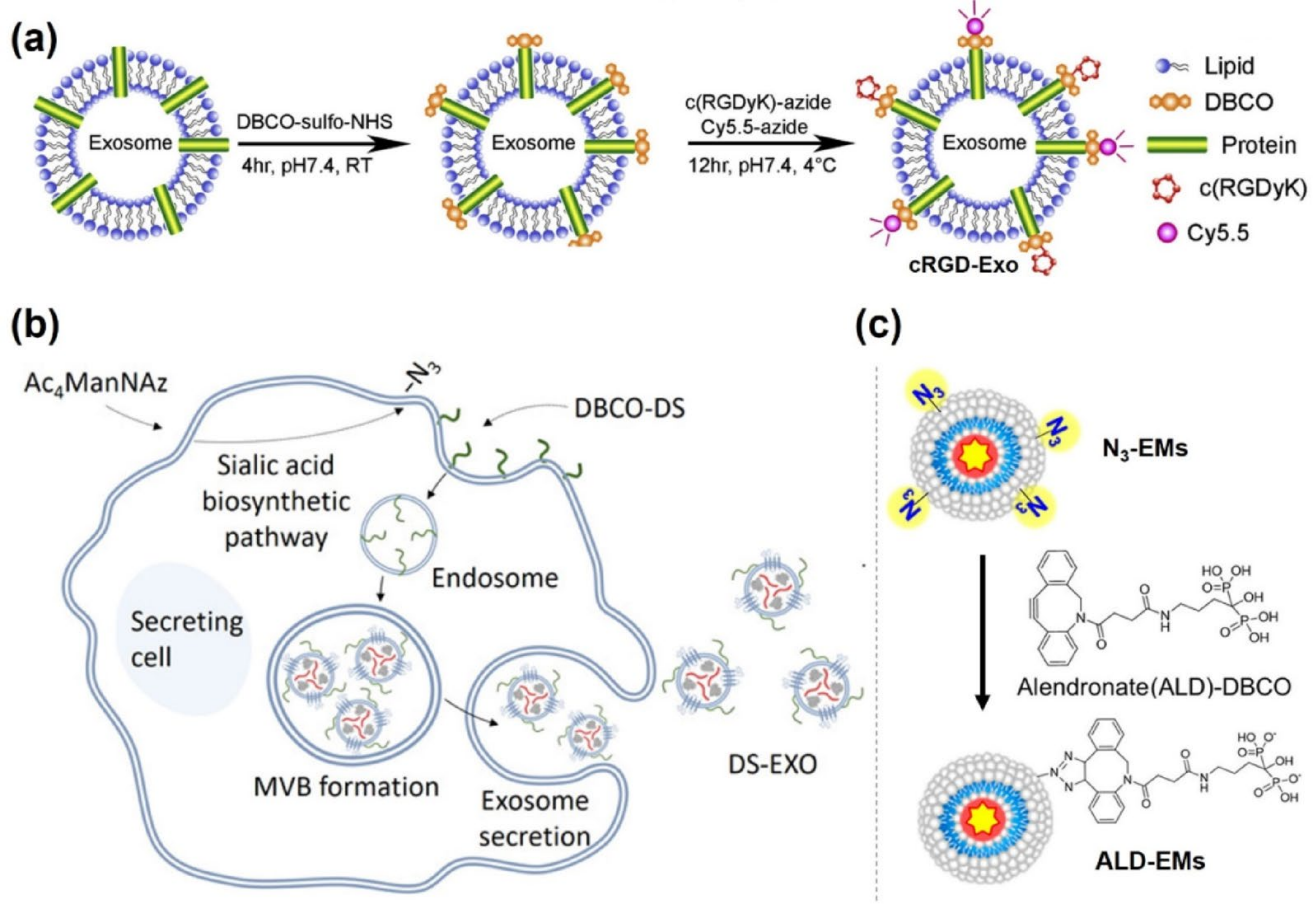
**a** Schematic illustration of cRGD-Exo synthesis. Reprinted from Ref. [157] with permission from Elsevier Inc, copyright 2018. **b** Schematic illustration of exosome surface engineering via MGE-mediated azide labeling and SPAAC-mediated DS functionalization. Reproduced from ref. [159], 2021, under the CC BY-NC 4.0 license. **c** Schematic illustration of ALD-EMs synthesis. Reprinted from Ref. [160] with permission from the American Chemical Society, copyright 2023

Cell-derived exosomes and nanovesicles are attractive nanomedicine platforms because they inherently provide a biologically derived membrane interface with distinct profiles, low immunogenicity, and effective protection of encapsulated cargo [146–151]. However, broader therapeutic use has often been limited by poor lesion selectivity after systemic administration, low functional density on the vesicle surface, and the difficulty of introducing additional surface functions without compromising vesicle integrity or recovery [152–155]. In this context, bioorthogonal chemistry has emerged as a useful surface-engineering strategy because it enables modular ligation under mild conditions, either by direct modification of isolated vesicles or by metabolic installation of clickable handles in donor cells before vesicle secretion. One of the earliest examples was reported by Anchordoquy et al., who established a proof-of-concept method for direct chemical modification of isolated exosomes [156]. In that study, surface-exposed amines on exosomal proteins were first reacted with 4-pentynoic acid via carbodiimide coupling to introduce terminal alkyne groups, and a model azide fluorophore was subsequently attached by CuAAC. Importantly, this early work showed that click-based conjugation could be performed without detectable changes in exosome size or in their adherence/internalization behavior toward recipient cells. A more translational step was reported by Gao et al., who used copper-free SPAAC to introduce a targeting ligand onto pre-isolated mesenchymal stem cells (MSC)-derived exosomes for ischemic brain therapy [157]. Purified exosomes were first reacted with DBCO-sulfo-NHS to couple DBCO to amine-bearing surface components and were then ligated with azide-functionalized c(RGDyK) peptide (Fig. 7a). The resulting cRGD-Exo acquired affinity for integrin αvβ3 expressed in vascular endothelial cells within ischemic brain lesions. Xie et al. reported M1 macrophage-derived exosomes conjugated with anti-CD47 and anti-SIRPα antibodies through pH-sensitive benzoic-imine linkers [158]. In this design, the azide groups were introduced to exosomes by metabolic incorporation of azide choline into membrane phospholipids at the donor-cell level, and the exosomes were subsequently isolated and functionalized with DBCO-labeled antibodies via SPAAC. Functionally, the antibody modules enabled tumor targeting and immune activation, whereas the native M1 exosomes re-educated tumor-associated macrophages toward an antitumor phenotype.

A further advance came when Park et al. used metabolic glycoengineering (MGE) to simplify exosome surface engineering at the donor-cell level [159]. MGE is the process of installing unnatural sugars, such as azide-bearing mannosamine (ManNAz), into surface glycans by leveraging endogenous glycan metabolic pathways. In this study, adipose-derived stem cells were first treated with acetylated ManNAz (Ac_4_ManNAz) to install azides via the sialic acid biosynthetic pathway, and the cell surface was then reacted with DBCO-conjugated dextran sulfate (DBCO-DS) (Fig. 7b). During exosome biogenesis, DS-decorated membrane components were inherited by the secreted vesicles, generating DS-EXOs that could be isolated by tangential flow filtration without additional post-isolation conjugation. The installed dextran sulfate served as a macrophage-targeting ligand for scavenger receptor A in inflamed joints. Compared with conventional exosome modification, this strategy better preserved yield and physicochemical characteristics, while markedly improving inflamed-joint accumulation and therapeutic efficacy in rheumatoid arthritis. This work was important because it shifted the field from direct exosome modification toward donor-cell-based preprogramming of exosome surfaces.

The same logic was subsequently extended to engineered exosome mimetics for bone-specific targeting. Lee et al. reported a scalable system in which MSCs were first metabolically labeled with Ac_4_ManNAz, and the resulting azide-bearing membrane was processed by extrusion to generate azide-displayed exosome mimetics (N_3_-EMs) [160]. A bone-targeting moiety, alendronate (ALD), was then introduced through DBCO-mediated click ligation to yield ALD-EMs (Fig. 7c). Although this system used exosome mimetics rather than naturally secreted exosomes, it is highly relevant because it demonstrates that the same bioorthogonal strategy can be transferred to more scalable nanovesicle analogs for regenerative medicine.

Wang et al. further generalized this donor-cell metabolic approach into a universal extracellular vesicle (EV) tagging platform [161]. By treating diverse parent cells with Ac_4_ManNAz, they generated azido-tagged EVs from cancer cells, MSCs, dendritic cells, and T cells, and showed that these vesicles could subsequently be functionalized with DBCO-cargo via click chemistry. A key feature of this work was the substantially higher density of chemical handles achieved by metabolic glycan labeling, with thousands of azides available per EV, enabling not only imaging and tracking but also high-density display of functional molecules.

A distinct conceptual advance was reported by Zhao et al., who developed a glycosylphosphatidylinositol (GPI)-based metabolic labeling strategy for exosome tracking and tropism screening [162]. Rather than relying on glycan engineering, they designed an azide-tagged phosphatidylinositol derivative (N_3_-Ino) that was metabolically converted into azide-tagged GPI and inserted into donor-cell membranes, enabling secretion of azide-labeled exosomes that could subsequently be labeled with DBCO-fluorophores.

Across these examples, bioorthogonal surface engineering can be organized into several recurring installation routes. Direct chemical introduction of clickable ligands, such as ligand exchange, silanization, or amide coupling, offers high chemical control over surface composition and conjugation sites, but requires careful consideration of colloidal stability, ligand orientation, and surface accessibility during post-synthetic functionalization and subsequent biological application. Amphiphilic polymer and lipid coatings provide a more general post-synthetic strategy for introducing bioorthogonal handles onto inorganic and hybrid nanomaterials, but coating density, spacer length, hydration, and interfacial packing should be optimized to ensure efficient conjugation and stable presentation of the installed functional modules. For cell-derived vesicles, donor-cell metabolic labeling or membrane engineering can preserve vesicle integrity and increase handle density, but batch-to-batch variability and off-target labeling must be carefully controlled. Thus, surface-level bioorthogonal engineering is most powerful when the clickable handle is treated not merely as a chemical tag, but as an interfacial design element whose density, orientation, hydration, and biological exposure are optimized together with the targeting, imaging, or therapeutic module.

## Molecular control of payload activation and release

**Fig. 8:**
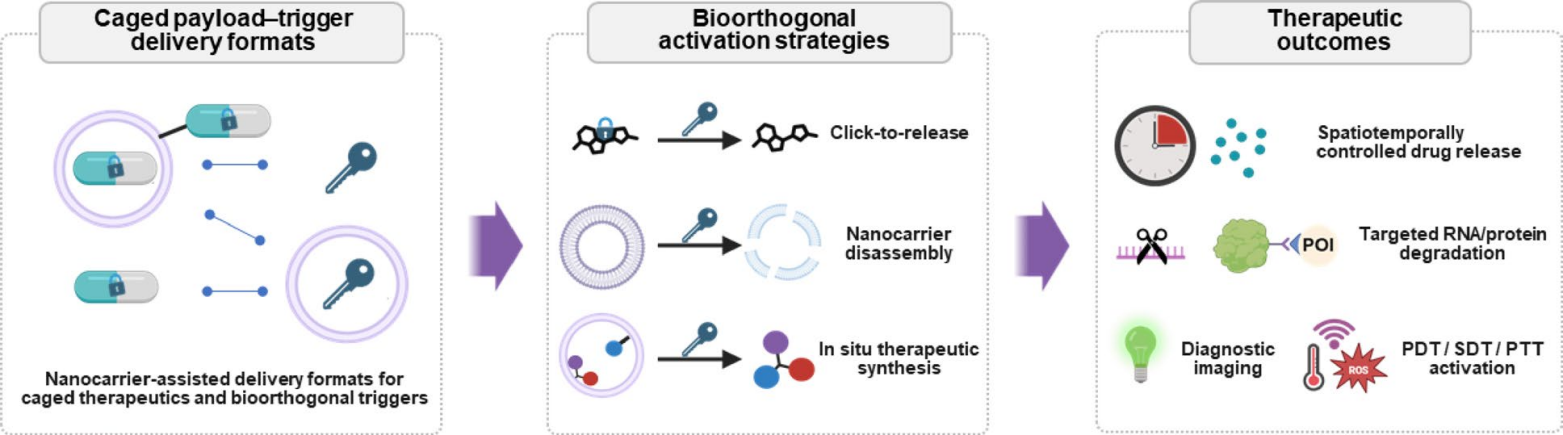
Representative strategies for molecular control of payload activation and release

Bioorthogonal chemistry has been explored in nanomedicine to address a major limitation of conventional nanocarriers, namely the limited control over when and where payloads become pharmacologically active. Rather than relying solely on carrier degradation or passive leakage, these strategies use orthogonal chemical reactions to directly control payload uncaging, release, or even in situ synthesis after nanomedicine delivery (Fig. 8, Table 3).

**Table 3:** Representative nanomedicine platforms for bioorthogonal payload activation

Reaction type	Caged drug	Trigger	Application	Refs
*Small-molecule anticancer payloads*				
IEDDA	Vinyl ether-linked PEG-b-Dox NP	Tetrazine	Chemotherapy	[163]
IEDDA	TCO-caged doxorubicin IONP	Tetrazine	Chemotherapy / FL imaging	[164]
IEDDA	TCO-linked doxorubicin/PAC-1 MNP	Tetrazine	Sequential chemotherapy / MR imaging	[165]
IEDDA	Vinyl ether-caged camptothecin liposome	NIR dye-linked tetrazine liposome	Chemotherapy / NIR FL imaging	[166]
IEDDA	Vinyl ether-caged camptothecin liposome	AuNR-PEG-Tz	Chemotherapy / MSOT imaging / PTT	[167]
IEDDA	TCO-caged doxorubicin	Tz-functionalized polymer micelle	Chemotherapy	[168]
IEDDA	TCO-caged doxorubicin micelle	Tz-loaded TME-responsive micelle	Chemotherapy	[169]
IEDDA	TCO-doxorubicin@ZIF-8	ICG@Tz-tk-PEG micelle	Chemotherapy	[170]
IEDDA	TCO-caged doxorubicin / TCO-caged hemicyanine	TZ@SWCNTs	Chemotherapy / NIR FL imaging	[171]
IEDDA	H₂S-responsive doxorubicin prodrug-loaded CONP	Trastuzumab-tetrazine	Chemotherapy / FL imaging	[172]
*Nucleic-acid & Degrader therapeutics*				
IEDDA	TCO-siRNA-immobilized IONP	Tetrazine	RNAi activation / gene silencing	[183]
IEDDA	Vinyl ether-caged MZ1-O@NP	Microneedle-delivered tetrazine	BRD4 degradation / chemotherapy	[189]
IEDDA	Split PROTAC precursor-loaded liposomes	Tetrazine / TCO precursor pair	In situ PROTAC assembly / BRD4 degradation	[190]
Staudinger reduction	Azide-caged SupTAC NP	THPP	BRD4 degradation	[191]
*Photoactivatable theranostic systems*				
IEDDA	Vinyl ether-caged CyPVE	Tetrazine-functionalized pH-responsive micelle	PDT / FL imaging	[194]
Staudinger reduction	Azide-caged methylene blue nanoplatform	Triphenylphosphine-based mitochondrial trigger	SDT / mitochondrial ROS generation	[196]
IEDDA	CyV/ZIF-90	TzCOF@Apt	PDT / FL imaging / tumor-targeted activation	[197]
Bioorthogonal in situ synthesis	TTB-NH₂@HA-PArg	Tumor-associated H₂O₂	PTT / photothermal imaging	[198]

### Small-molecule anticancer payloads

**Fig. 9:**
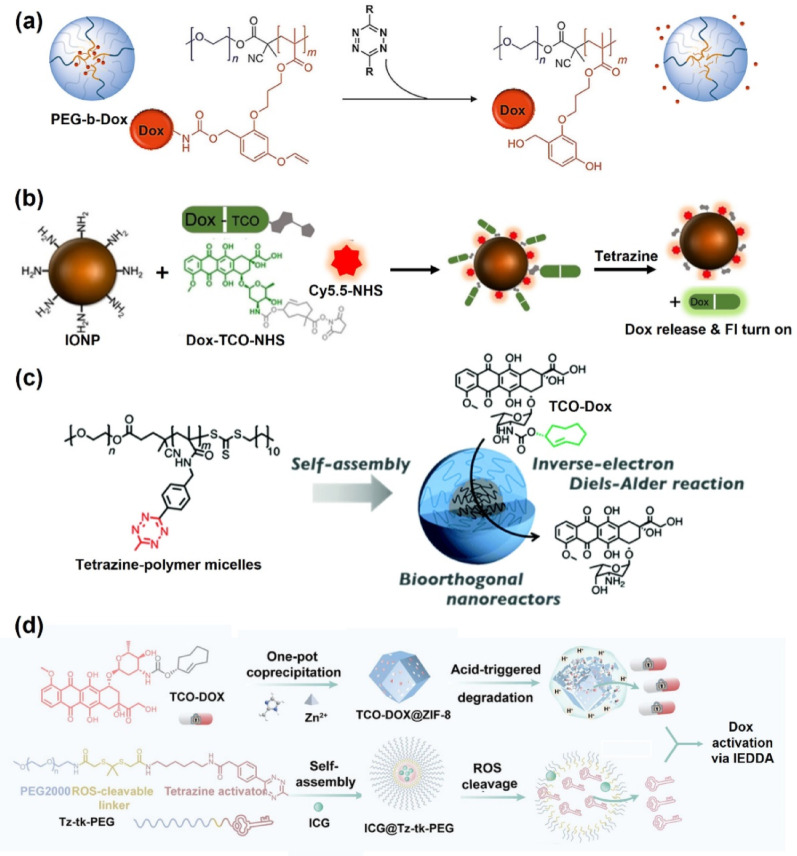
**a** Tetrazine-mediated Dox release from amphiphilic block-co-polymer (PEG-b-Dox) via IEDDA reaction. Reprinted from Ref. [163] with permission from Wiley–VCH, copyright 2017. **b** Schematic illustration of Dox-TCO conjugated IONPs and tetrazine-mediated Dox release. Reprinted from Ref. [164] with permission from Wiley–VCH, copyright 2020. **c** Tetrazine-functionalized block copolymers as nanoreactors for IEDDA-mediated conversion of Dox-TCO into Dox. Reprinted from ref. [168] with permission from The Royal Society of Chemistry, copyright 2022. **d** Schematic illustration of the preparation of pH-responsive TCO-DOX@ZIF-8 and ROS-responsive ICG@Tz-tk-PEG and the subsequent IEDDA-mediated activation of Dox. Reprinted from Ref. [170] with permission from Wiley–VCH, copyright 2025

Bradley et al. reported one of the earliest examples, a tetrazine-responsive polymeric nanodrug (PEG-b-Dox) in which the anticancer drug doxorubicin (Dox) was covalently incorporated into self-assembled PEG-based NPs through a vinyl ether-containing self-immolative linker (Fig. 9a) [163]. In this system, PEG-*b*-Dox remained weakly cytotoxic until exposure to tetrazine, which triggered the IEDDA reaction between tetrazine and the vinyl ether, followed by linker fragmentation to restore Dox activity. This study established the basic concept that bioorthogonal chemistry can provide payload-level control for on-demand drug release.

Using a similar principle, Yigit et al. reported tetrazine-triggered Dox release system based on IONP core. In this platform, Dox was conjugated to dextran-coated IONPs through a *trans*-cyclooctene-NHS linker (Dox-TCO-NHS) (Fig. 9b) [164]. Notably, the intrinsic fluorescence of doxorubicin was quenched by direct conjugation to the TCO linker; thus, tetrazine not only controlled drug release but also restored fluorescence in a concentration-dependent manner, enabling a self-reporting theragnostic system. In a related study, the same group further showed that two small-molecule payloads, Dox and procaspase activating compound 1 (PAC-1), could be installed through TCO linkers with different release kinetics, allowing a single tetrazine trigger to generate sequential dual-payload activation [165].

This release logic was soon extended from cell-based proof-of-concept studies to in vivo theranostic nanosystems. Wu et al. reported a two-component liposomal bioorthogonal nanosystem in which one liposome encapsulated a vinyl ether–caged camptothecin prodrug and the other carried a tetrazine-linked NIR fluorophore [166]. The tetrazine–vinyl ether reaction simultaneously released camptothecin and restored NIR fluorescence, enabling parallel readouts of drug activation and imaging. In HeLa xenografts, the combined treatment produced strong tumor-selective fluorescence and suppressed tumor growth more effectively than either component alone, even outperforming free camptothecin, while body weight remained stable. In a follow-up study, the same group replaced the fluorescent trigger carrier with a multifunctional gold nanorod-based nanotrigger [167]. In this system, PEGylated tetrazine-functionalized gold nanorods (AuNR-PEG-Tz) were paired with a liposomal vinyl ether–caged camptothecin prodrug (LIP-VE-CPT). Here, AuNRs served as the tetrazine trigger carrier, a multispectral optoacoustic tomography (MSOT) contrast agent, and a photothermal transducer. In PBS (pH 7.4), camptothecin release exceeded 85% at 48 h in the presence of AuNR-PEG-Tz, whereas only ~ 10% was released without the trigger. In vivo, 3D MSOT showed strong tumor localization of AuNR-PEG-Tz, and the combination of bioorthogonal release and photothermal heating led to markedly improved tumor inhibition. This study broadened the payload-control concept by embedding the cleavage reaction within a multifunctional inorganic trigger platform.

Another important direction is the development of bioorthogonal nanoreactors, in which the carrier itself is engineered to promote the activation reaction. Nishimura et al. reported tetrazine-functionalized micelles that served as confined reaction compartments for the activation of TCO-caged Dox (Fig. 9c) [168]. Formed through the self-assembly of tetrazine-bearing amphiphilic block copolymers, these polymeric micelles provided a hydrophobic interior that increased the local effective concentration and interactions between the reactive partners. Consequently, the micellar platform accelerated prodrug activation relative to free tetrazine amine, although the magnitude of enhancement depended on the stereochemistry of the TCO–Dox prodrug. A related but more spatially controlled approach was demonstrated by Xie et al., who reported two tumor-responsive polymer micelles to separately deliver a TCO-Dox prodrug and a tetrazine activator, such that uncaging of Dox via the IEDDA reaction occurred preferentially at the intersection of tumor-associated acidic and enzymatic conditions [169]. More recently, Hao et al. advanced this concept by integrating pH- and NIR-responsive nanoplatforms (Fig. 9d) [170]. In their system, TCO-caged Dox was loaded into zeolitic imidazolate framework-8 (ZIF-8), which degrades in the acidic tumor microenvironment. In parallel, the tetrazine activator was conjugated to a nanomicelle through a thioketal linker, and the micelle also encapsulated the photosensitizer indocyanine green (ICG). Following tumor accumulation via the EPR effect, ZIF-8 released TCO-DOX through acid-triggered degradation, whereas NIR irradiation stimulated ICG to generate reactive oxygen species (ROS), which in turn cleaved the thioketal linker to release tetrazine. This system illustrates how bioorthogonal chemistry can be integrated with stimuli-responsive nanotechnology to enable spatiotemporally gated chemotherapy with reduced systemic toxicity.

**Fig. 10:**
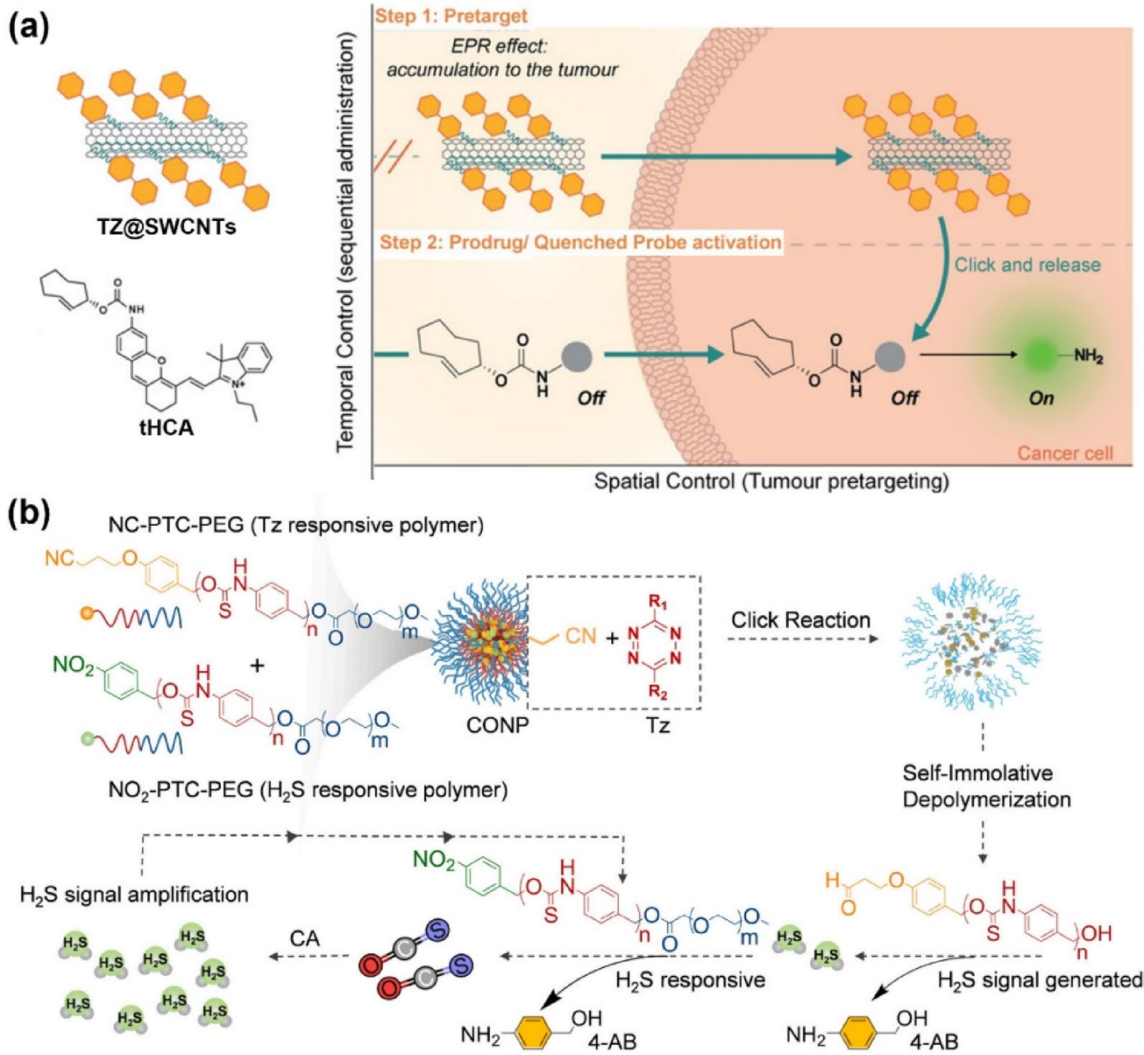
**a** Schematic illustration of pretargeted tumor-selective activation strategy based on EPR-driven accumulation of TZ@SWCNTs and subsequent tetrazine–TCO IEDDA cleavage, enabling local release of active drugs or imaging probes at the tumor site. Reprinted from Ref. [171] with permission from Wiley–VCH, copyright 2020. **b** Tetrazine-triggered bioorthogonal activation of CONP nanoparticle disassembly and H_2_S production. Reprinted from Ref. [172] with permission from Elsevier Inc, copyright 2025

Bernardes et al. reported a pretargeting-based bioorthogonal activation strategy for tumor-localized drug delivery, in which tetrazine-functionalized single-walled carbon nanotubes (TZ@SWCNTs) were first accumulated at the tumor site, followed by administration of TCO-caged fluorophores (tHCA) or TCO-Dox prodrugs as second-step substrates (Fig. 10a) [171]. In this system, IEDDA-mediated click-to-release enabled local conversion of the administered substrates into active imaging or therapeutic agents only at sites containing the prelocalized nanotubes. Extending this concept, Yuan et al. employed tetrazine-conjugated trastuzumab as a pretargeting agent for HER2-positive tumors to initiate tumor-localized depolymerization of a self-assembled nanocarrier composed of a tetrazine-responsive polymer (NC-PTC-PEG) and an H_2_S-responsive polymer (NO_2_-PTC-PEG) (Fig. 10b) [172]. In this platform, the 3-isocyanopropyl group of NC-PTC-PEG first reacted with tetrazine to generate carbonyl sulfide, which was rapidly converted to H_2_S in vivo. The resulting H_2_S signal was then amplified through degradation of NO_2_-PTC-PEG, ultimately leading to activation of an H_2_S-responsive prodrug. This strategy achieved potent tumor inhibition across heterogeneous HER2-positive tumor models and highlights how bioorthogonal nanomedicine can be coupled with signal conversion and amplification to address limitations associated with variable antigen expression.

**Fig. 11:**
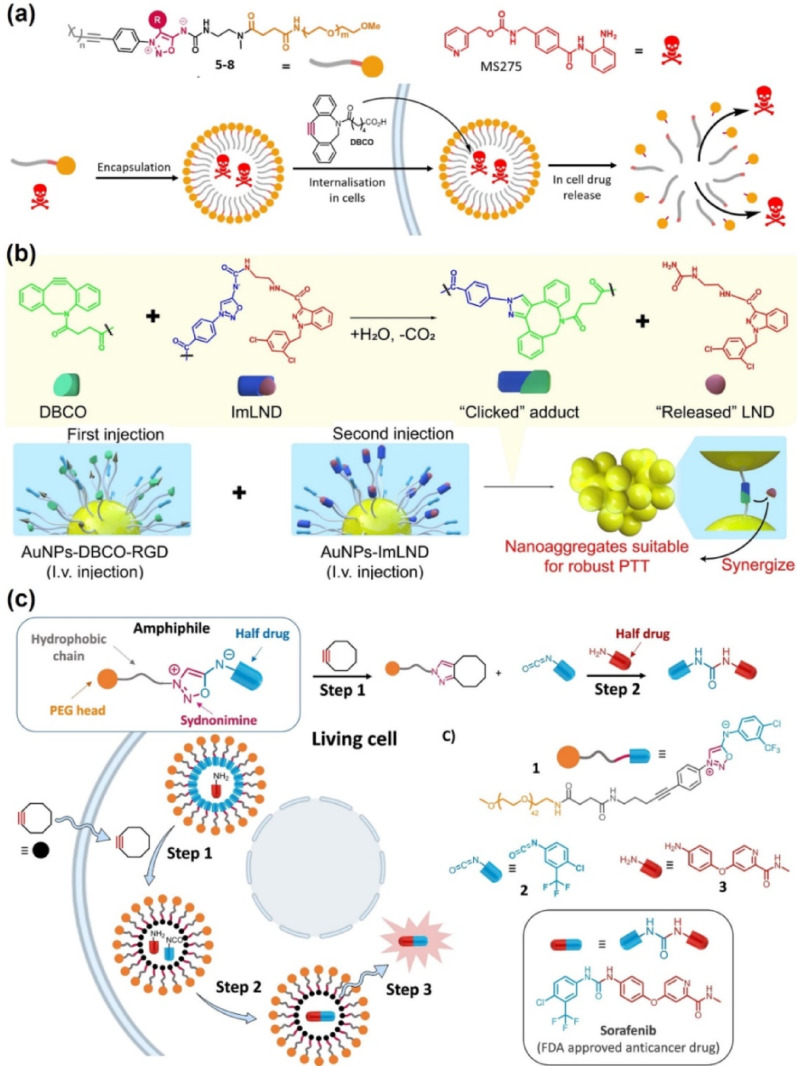
**a** Schematic illustration of DBCO-induced payload release from sydnonimine-based lipid micelles through the SPSIC reaction. Reprinted from Ref. [174] with permission from Wiley–VCH, copyright 2023. **b** SPSIC-triggered drug release upon reaction of DBCO-functionalized AuNPs with sydnonimine-caged drug-conjugated AuNPs. Reprinted from Ref. [175] with permission from Wiley–VCH, copyright 2024. **c** Intracellular operation of stimuli-responsive micelles: SPSIC-mediated activation, confined drug formation, and subsequent drug diffusion. Reprinted from Ref. [179] with permission from Wiley–VCH, copyright 2025

Beyond tetrazine-based systems, SPSIC chemistry has also been explored to expand the toolbox for regulating small-molecule payloads. Taran et al. demonstrated SPSIC-mediated control over drug release from micellar nanomedicine platforms [173, 174]. In this strategy, an amphiphilic monomer (M5-8) was designed by linking a hydrophobic tail and a PEG head through a sydnonimine spacer (Fig. 11a). The resulting amphiphilic monomers spontaneously self-assembled into micelles capable of encapsulating hydrophobic anticancer drugs (MS275). Upon reaction with DBCO, the sydnonimine-based micelles underwent rapid disassembly, leading to burst release of the loaded payloads. In a related example, Zhang et al. further used the SPSIC reaction to control drug release in a two-component nanoplatform [175]. In this system, two PEGylated AuNPs were prepared: AuNPs-DBCO-RGD incorporated an RGD peptide for tumor targeting through integrin αvβ3 binding, as well as a DBCO moiety to trigger SPSIC-mediated drug release, whereas AuNPs-ImLND carried an iminosydnone-caged lonidamine prodrug (Fig. 11b). Using a pre-targeting strategy, AuNPs-DBCO-RGD were first administered to tumor-bearing mice, followed by injection of AuNPs-ImLND. The subsequent reaction between the iminosydnone group and the DBCO handle induced bioorthogonal click-and-release, which simultaneously liberated lonidamine.

Beyond bioorthogonal click-to-release reactions, bioorthogonal ligation reactions have emerged as an alternative strategy for the in situ generation of active therapeutics from non-toxic precursors. Early efforts primarily relied on CuAAC and other transition metal-catalyzed transformations, establishing a foundation for reaction-enabled drug synthesis in living systems [176–178]. More recently, the Taran group developed micellar nanoreactors for the metal-free synthesis of small-molecule therapeutics from encapsulated precursors via the SPSIC reaction (Fig. 11c) [179]. In this system, sydnonimine-based amphiphiles bearing one sorafenib precursor fragment self-assembled into micelles that encapsulated the complementary precursor. Following sequential administration of the micelles and a DBCO trigger to living cancer cells, SPSIC-mediated release of a reactive intermediate occurred within the micellar core, enabling subsequent ligation with the second precursor to form active sorafenib. This work highlights the potential of bioorthogonal nanomedicine to move beyond conventional uncaging and toward in situ construction of therapeutically active small molecules.

### Nucleic-acid and degrader therapeutics

Compared with small-molecule payloads, the application of bioorthogonal chemistry to other therapeutic modalities, including nucleic acids and protein degraders, remains relatively limited. Nevertheless, these studies highlight several important directions for the development of programmable nanomedicine.

**Fig. 12:**
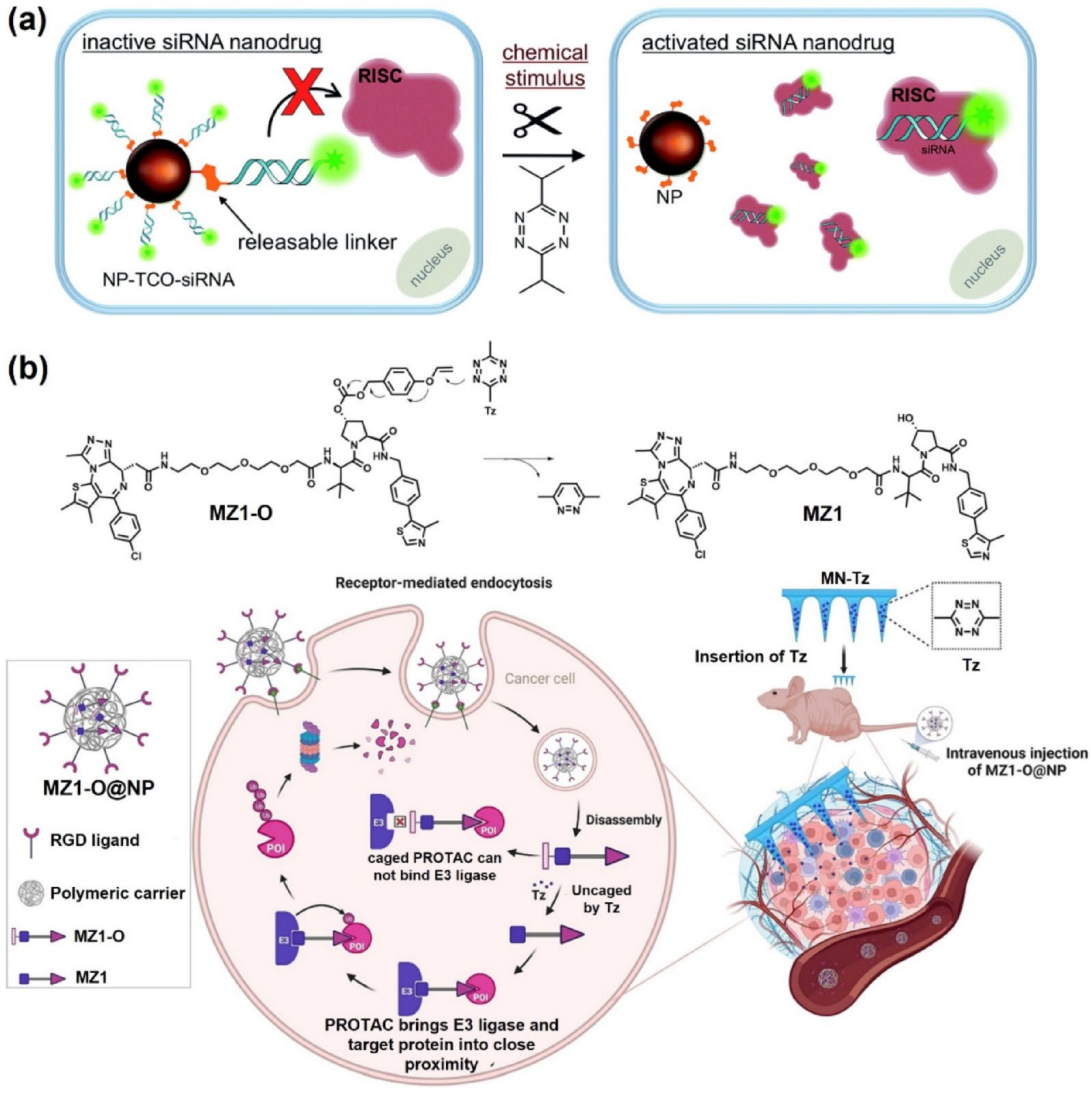
**a** Schematic representation of IEDDA-triggered activation of NP-TCO-siRNA in vitro. Reprinted from ref. [183] with permission from The Royal Society of Chemistry, copyright 2017. **b** Schematic illustration of tumor-targeted delivery of MZ1-O by a polymeric carrier and its tetrazine-triggered activation by a microneedle patch, leading to BRD4 degradation in cancer cells. Reprinted from Ref. [189] with permission from Elsevier Inc, copyright 2023

Small interfering RNA (siRNA) therapeutics are a class of drugs that induce sequence-specific gene silencing via the RNA interference (RNAi) pathway [180]. Owing to their high sequence specificity and applicability to otherwise undruggable targets, siRNA have attracted considerable interest; however, precise temporal control over their activation remains challenging [181, 182]. Royzen et al. showed that bioorthogonal click-to-release chemistry can provide on-demand control over siRNA function by immobilizing siRNA on dextran-coated iron oxide NPs through a tetrazine-cleavable linker [183]. In this system, steric hindrance imposed by the NP scaffold prevented access of the RNA-processing machinery, thereby maintaining the siRNA payload in an inactive state until intracellular bioorthogonal uncaging occurred (Fig. 12a). This work demonstrates the potential of bioorthogonal chemistry to enable temporally regulated RNAi activation and precise control of gene expression.

Proteolysis-targeting chimeras (PROTACs) are bifunctional degraders that selectively eliminate proteins of interest by bringing them into proximity with the cellular ubiquitin–proteasome system [184, 185]. PROTACs are promising therapeutic agents because they induce event-driven and catalytic degradation of target proteins. However, their therapeutic potential is often limited by poor pharmacokinetic profiles, limited cell permeability, and undesirable off-target effects [186–188]. To overcome these limitations, recent studies have explored nanoplatforms that encapsulate caged PROTACs, enabling degrader activation via bioorthogonal chemistry. For example, Ping et al. developed a bioorthogonally activatable PROTAC prodrug for site-specific cancer therapy [189]. In this study, the BRD4 degrader MZ1 was caged with a moiety that could be removed by a tetrazine-mediated IEDDA bond-cleavage reaction (Fig. 12b). For delivery, MZ1-O was encapsulated in RGD-functionalized PLGA NPs (MZ1-O@NP), whereas tetrazine was administered locally using a dissolvable microneedle patch (MN-Tz). This design was intended to suppress PROTAC activity during systemic circulation and restore it selectively at the tumor site. In melanoma and A549 tumor models, the combination of MZ1-O@NP and MN-Tz produced marked tumor growth inhibition without significant body weight loss, indicating that bond-cleavage bioorthogonal chemistry can provide spatially selective activation of PROTACs in vivo.

**Fig. 13:**
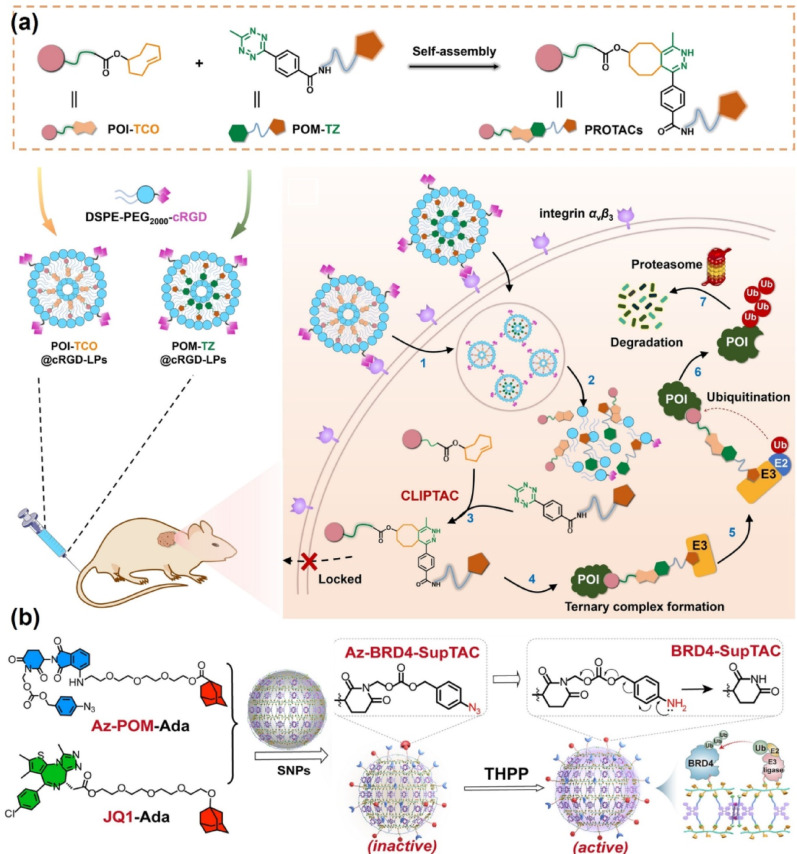
**a** Scheme of the Nano-CLIPTAC strategy for selective degradation of tumor-associated proteins. Reprinted from Ref. [190] with permission from Wiley–VCH, copyright 2025. **b** Schematic illustration of bioorthogonally controlled BRD4 degradation using Az-BRD4-SupTAC. Reprinted from Ref. [191] with permission from Elsevier Inc, copyright 2026

In a conceptually distinct strategy, Xu et al. developed Nano-CLIPTACs, in which two smaller PROTAC precursors were separately delivered by cRGD-modified liposomes and subsequently underwent in situ tetrazine–TCO ligation to generate the active PROTACs only after tumor-directed delivery (Fig. 13a) [190]. Rather than uncaging a preassembled degrader, this system relies on localized bioorthogonal bond formation to construct the PROTAC directly at the disease site. This approach is particularly attractive because it may alleviate the intrinsic hook effect associated with conventional PROTACs by restricting formation of the active degrader to regions where both precursor components are locally enriched.

In a more recent study, Wang et al. introduced SupTACs as self-assembled supramolecular NPs for tissue-specific protein degradation [191]. This platform was generated by self-assembly of adamantane-functionalized metal–organic cages and β-cyclodextrin-conjugated polyethyleneimine, followed by surface incorporation of E3 ligase recruiters (JQ1-Ada) and chemically caged BRD4-binding ligands (Az-POM-Ada), which could be activated through bioorthogonal Staudinger reduction (Fig. 13b). In the absence of THPP, the trigger for the Staudinger reaction, the caged SupTAC exhibited minimal BRD4 degradation. By contrast, THPP treatment restored degrader activity, resulting in approximately 60% BRD4 reduction in cells. In vivo, the combination of lung-targeted Az-BRD4-SupTAC and THPP induced more than 60% BRD4 degradation in lung tissue, with no evident degradation in non-pulmonary organs. This study demonstrates that bioorthogonal chemistry can be exploited to achieve tissue-specific and temporally controlled activation of supramolecular degrader nanomedicine.

### Photoactivatable theranostic systems

Bioorthogonal chemistry has also been applied to nanomedicines carrying photodynamic, photothermal, or sonodynamic agents. The primary objective is to keep the imaging or therapeutic functions inactive during circulation and restore them only after tumor localization, thereby reducing off-target toxicity. In this context, bioorthogonal chemistry can also provide real-time optical reporting of activation and a means to synchronize therapeutic function with the availability of local triggers.

Photodynamic therapy (PDT) relies on light-triggered ROS generation by photosensitizers, but its efficacy is often limited by inadequate target specificity and dark toxicity [192, 193]. To address these limitations, Wang et al. developed a macrotheranostic prodrug activation strategy that combined the acidic tumor microenvironment with bioorthogonal chemistry for precise PDT [194]. Their platform consisted of a vinyl ether-caged near-infrared hemicyanine macroprodrug (CyPVE) and a tetrazine-functionalized pH-responsive polymer that formed acid-sensitive micelles. Under acidic tumor conditions, micellar disassembly exposed tetrazine groups, which subsequently decaged CyPVE and restored both fluorescence and ROS-generating activity. Using a related concept for sonodynamic therapy (SDT)—in which ultrasound activates sonosensitizers to generate ROS for target elimination [195]—Zhang et al. reported a mitochondria-specific bioorthogonal activation system based on the Staudinger reaction [196]. In this system, mitochondrial colocalization of a triphenylphosphine-based trigger and a caged methylene blue nanoplatform enabled organelle-specific release of the active sonosensitizer, thereby allowing programmable ultrasound-triggered ROS generation. This work is notable because it introduces organelle-specific bioorthogonal activation into sonodynamic nanomedicine and offers a more tunable alternative to endogenous activation mechanisms.

**Fig. 14:**
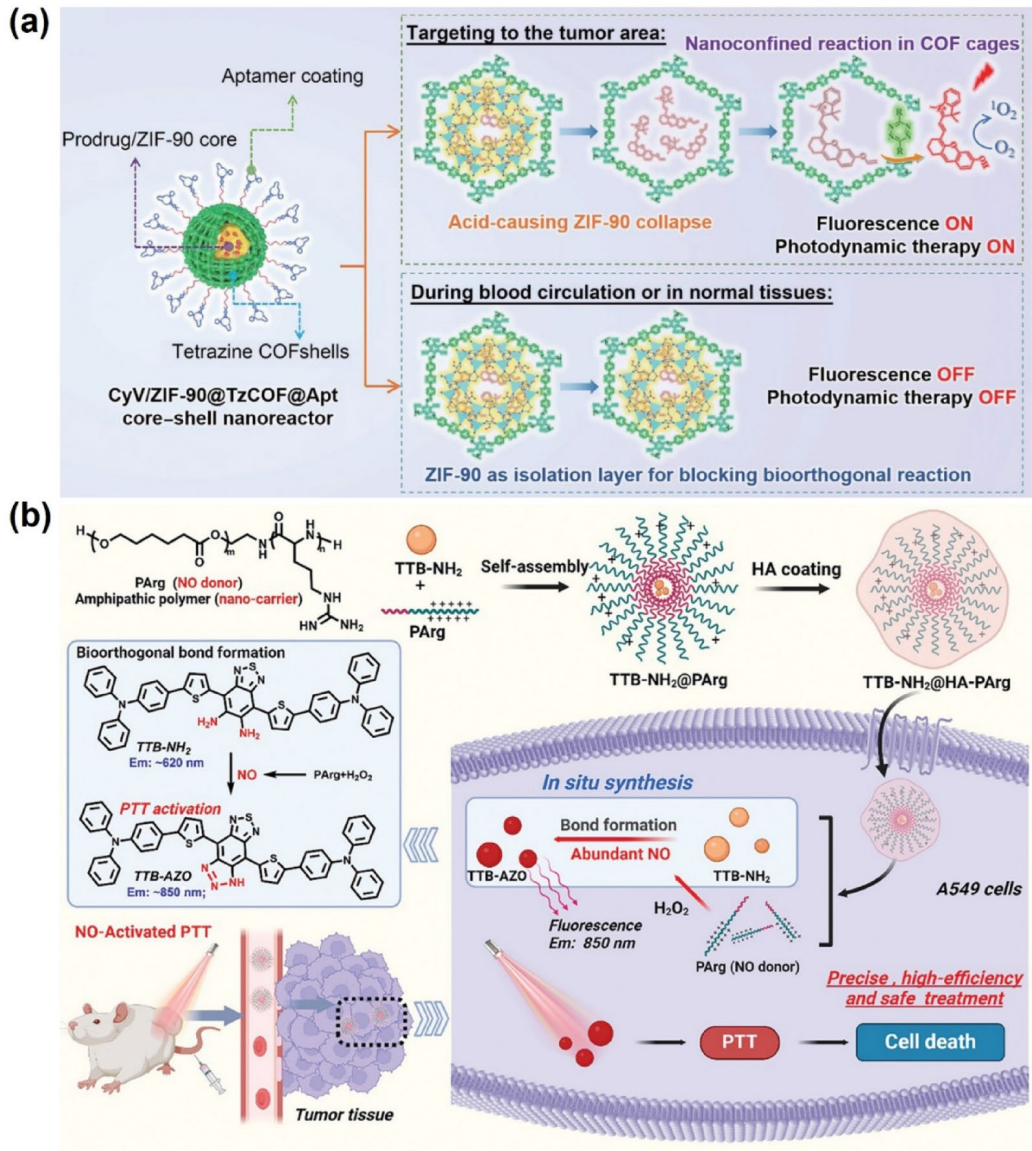
**a** Activation mechanism of CyV/ZIF-90@TzCOF@Apt in the acidic tumor microenvironment, leading to IEDDA-mediated photosensitizer decaging. Reprinted from Ref. [197] with permission from Springer Nature, copyright 2022. **b** Activation mechanism of TTB-NH_2_@HA-PArg under tumor-associated H_2_O_2_, leading to in situ bioorthogonal formation of the photothermal agent. Reprinted from Ref. [198] with permission from Wiley–VCH, copyright 2024

Zhang et al. reported a more spatially coordinated strategy: a nanovoid-confinement and click-activated nanoreactor for photodynamic therapy [197]. Their platform (CyV/ZIF-90@TzCOF@Apt) combined an acid-sensitive ZIF-90 core carrying vinyl ether-caged near-infrared hemicyanine (CyV) with a tetrazine-based covalent organic framework shell that served as the bioorthogonal trigger layer, together with aptamer–polymer modification for tumor targeting (Fig. 14a). In this system, the ZIF-90 core prevented premature activation during circulation but disassembled in the acidic tumor microenvironment, enabling a nanoconfined bioorthogonal reaction between CyV and the tetrazine-rich shell. By synchronizing trigger and prodrug delivery within a defined nanospace, this nanoreactor enhanced activation efficiency, improved tumor inhibition, and reduced off-target toxicity. More recently, Liu et al. extended this concept to photothermal therapy by developing a nitric oxide-responsive bioorthogonal codelivery nanoassembly for in situ synthesis of a photothermal agent (Fig. 14b) [198]. Their TTB-NH_2_@HA-PArg platform used poly(L-arginine) as both a nanocarrier and an H_2_O_2_-responsive nitric oxide donor, enabling tumor-associated H_2_O_2_ to trigger conversion of TTB-NH_2_ into the active photothermal agent TTB-AZO. This transformation was accompanied by a fluorescence shift to ~ 850 nm, allowing real-time monitoring of activation. Collectively, these studies demonstrate that bioorthogonal nanomedicine can be extended beyond prodrug activation to spatially confined or stimulus-coupled in situ generation of photoactive therapeutics.

Collectively, the studies discussed in this section demonstrate that bioorthogonal chemistry can regulate nanomedicine function by controlling payload activation, release, or in situ synthesis. Most reported systems rely on two-component designs, in which a bioorthogonal trigger and a caged payload or precursor-bearing nanoplatform must encounter each other in complex biological environments. Thus, reaction efficiency depends not only on intrinsic reaction kinetics, but also on the time-dependent concentration, pharmacokinetics, tissue penetration, cellular uptake, and subcellular localization of each component. Although many studies have demonstrated bioorthogonal activation in cellular and in vivo models, future platforms should more rigorously evaluate trigger–payload encounters across cellular, tissue, and organismal scales. These analyses should clarify whether both components reach the same cells and compatible subcellular compartments, how tumor penetration, extracellular matrix barriers, local retention, and microenvironmental conditions affect spatial overlap, and how biodistribution, clearance, circulation half-life, and dosing interval determine temporal coordination in vivo. Such multiscale insights will be essential for moving bioorthogonal payload control beyond proof-of-concept activation toward truly programmable and precise nanomedicine with greater translational potential.

## Bioorthogonal programming beyond single NPs

Because bioorthogonal reactions can proceed selectively within complex biological environments, they can be used to program interactions among nanoparticles, cells, and disease-associated tissues after administration. In this context, bioorthogonal chemistry provides a means to spatially organize nanomedicines in vivo, chemically define target sites, and generate higher-order structures or cell-associated assemblies that are not present in the original formulation.

### Bioorthogonal pre-targeting of disease-localized nanomedicines for imaging and therapy

**Fig. 15:**
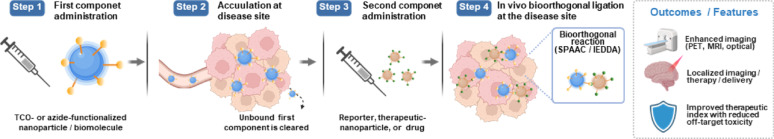
Schematic illustration of bioorthogonal pre-targeting strategy for disease-localized nanomedicine

Bioorthogonal pre-targeting offers a conceptually distinct solution to a central limitation of NP imaging and therapy: the mismatch between the slow pharmacokinetics of nanomedicines and the short half-lives or off-target liabilities of imaging and therapeutic reporters. In this strategy, a disease-localized NP or other targeting vector is administered first and allowed to accumulate at the site of interest, typically through the EPR effect or a disease-associated retention process (Fig. 15). A complementary imaging probe, radionuclide-bearing ligand, or secondary NP is then administered as a second step and covalently linked in vivo through a bioorthogonal reaction. Although the core logic of pre-targeting is conserved across studies, its biological scope has expanded substantially over time; therefore, several representative examples are discussed in this chapter (Table 4).

**Table 4:** Representative bioorthogonal pre-targeting systems for imaging and therapy

First component	Second component	Reaction type	Reaction site	Functional outcome	Refs
DBCO-PEG-MSNs	^18^F-azide reporter	SPAAC	Tumor region	Pretargeted PET imaging	[199]
Fe₃O₄@TCO	^177^Lu-DOTA-Tz	IEDDA	Tumor interstitium	In vivo radiolabeling / reduced liver radiation	[200]
TCO-cPLANPs	^131^I-labeled tetrazine	IEDDA	PSMA-positive tumor	SPECT imaging / radiotherapy	[201]
DBCO-loaded pretargeted NPs	Azide-POLY-PROTAC NPs	SPAAC	Tumor region	BRD4 degradation / PDT-combined therapy	[202]
pHLIP-Tz	^18^F-TCO-liposomes	IEDDA	Acidic tumor membrane	Pretargeted PET imaging / liposome anchoring	[203]
pHLIP-Tz	TCO-HSA-ICG NPs	IEDDA	Acidic tumor membrane	FL imaging / PTT	[204]
TCO-anti-CD11b antibody	DOX-loaded MSNs-Tz	IEDDA	CD11b⁺ myeloid cells	Deep-tumor chemotherapy	[205]
DSPE-PEG-N₃	Rap@PAG-DBCO	SPAAC	Tumor-draining lymph nodes	Rapamycin delivery / αPD-L1 immunotherapy enhancement	[206]
HA/DBCO-Au:Ag₂Te QDs	NK92-N₃ cells	SPAAC	CD44-positive tumor	NK-cell recruitment / tumor cell killing	[207]
sphNP-TCO	⁶⁸Ga-IONP-Tz	IEDDA	Atherosclerotic plaques	Pretargeted plaque PET imaging	[208]
ACRA-TCO nanobubbles	mTz-LNP-Cy5 / mTz-mRNA-LNP	IEDDA	FUS-treated tissue	Targeted mRNA-LNP delivery / FL imaging	[209]

**Fig. 16:**
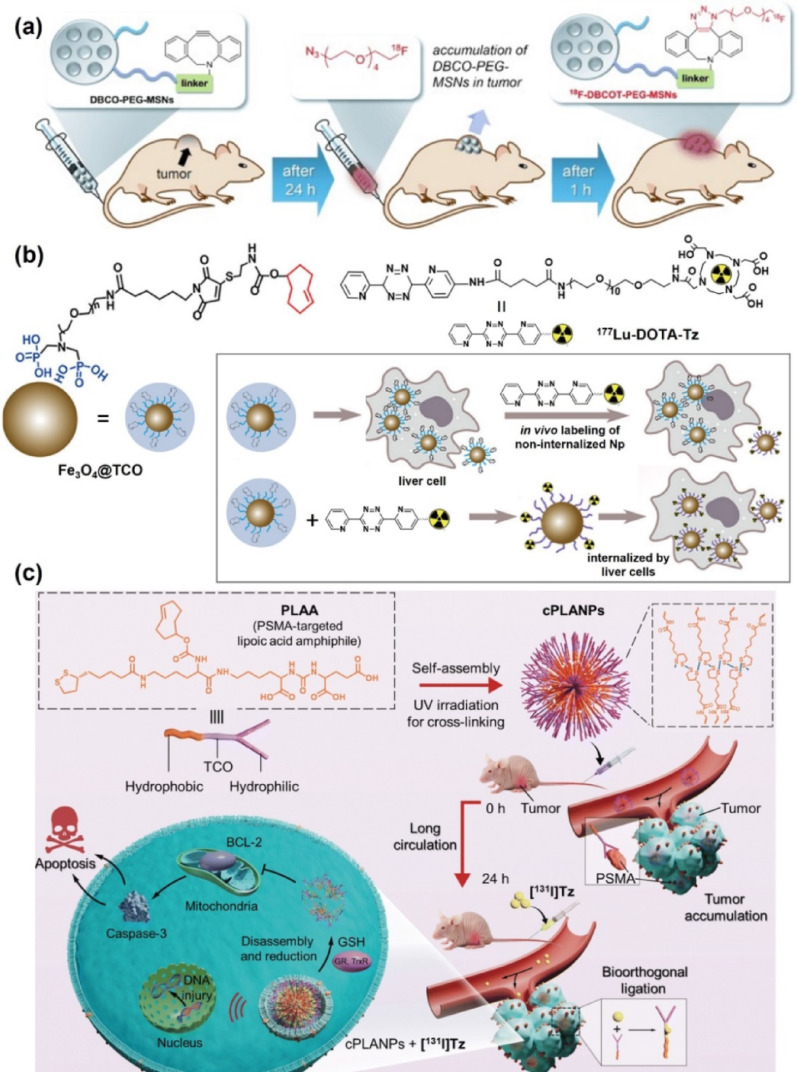
**a** In vivo radiolabeling of tumor-localized mesoporous silica nanoparticles with an ^18^F-labeled azide probe for PET imaging Reprinted from Ref. [199] with permission from Wiley–VCH, copyright 2013. **b** Schematic illustration of a pre-targeted radiotherapy strategy based on TCO-functionalized iron oxide nanoparticles, designed to minimize hepatic radiation exposure. Adapted from Ref. [200] with permission from Wiley–VCH, copyright 2026. **c** Schematic overview of the preparation of cross-linked, PSMA-targeted lipoic acid nanoparticles (cPLANPs) and their pre-targeted radiotherapeutic mechanism. Reprinted from ref. [201] with permission from The Royal Society of Chemistry, copyright 2024

An early proof-of-concept for bioorthogonal pre-targeting with nanomaterials was reported by Kim et al., who demonstrated MSN-based pre-targeting for PET imaging via SPAAC chemistry [199]. In this study, DBCO-conjugated MSNs (DBCO-PEG-MSNs) were first allowed to accumulate in tumors via the EPR effect, followed by administration of a small ^18^F-azide reporter with a short half-life (^18^F, 109.8 min) to generate radiolabeled MSNs in vivo (Fig. 16a). Pre-targeted mice exhibited markedly stronger tumor PET signals than mice receiving the radiotracer alone, and the imaging effect increased in a dose-dependent manner with the amount of preinjected MSNs. A conceptually similar nanoradiotherapeutic strategy was reported by Gao et al., who developed a tumor selective radiolabeling strategy to reduce hepatic radiation exposure from radiolabeled NPs [200]. Their nanosystem consisted of TCO-functionalized iron oxide NPs (Fe_3_O_4_@TCO) and a hydrophilic ^177^Lu-DOTA-Tz probe designed to remain extracellular and selectively react only with NPs that were still accessible within the tumor interstitium (Fig. 16b). The rationale leveraged the distinct subcellular fates of NPs in tumors and liver: in tumors, a substantial fraction remained extracellular and accessible, whereas in the liver NPs were rapidly internalized by resident cells and became inaccessible to the hydrophilic tetrazine probe. In subcutaneous tumor models, this two-step strategy markedly reduced liver signal compared with directly pre-radiolabeled NPs and increased the tumor-to-liver ratio by nearly threefold. Although absolute tumor uptake remained modest, this work is noteworthy because it reframed pre-targeting as a means of exploiting organ-specific subcellular accessibility, providing a biologically informed strategy for safer radiolabeled nanotheranostics.

Rather than relying solely on passive, EPR-mediated tumor accumulation, Wu et al. reported tumor-targeted cross-linked lipoic acid nanoparticles (cPLANPs) for pre-targeted SPECT imaging and radiotherapy (Fig. 16c) [201]. In this platform, the cPLANPs integrated prostate-specific membrane antigen (PSMA)-mediated tumor targeting, intrinsic antitumor activity derived from the lipoic acid-based core, and TCO moieties for subsequent bioorthogonal labeling. Following PSMA-directed tumor accumulation of cPLANPs, administration of ^131^I-labeled tetrazine enabled in vivo labeling of the cPLANPs through IEDDA chemistry. The resulting platform achieved strong tumor inhibition with minimal off-target toxicity, illustrating how bioorthogonal pre-targeting can be incorporated into a synergistic nanoradiotherapy system. Another therapeutic expansion of this concept was reported by Yu et al., who engineered tumor-environment-responsive polymeric PROTAC NPs that could be captured by pre-targeted NPs via SPAAC ligation, thereby amplifying intratumoral PROTAC release through sequential reaction with extracellular MMP-2 and intracellular GSH [202]. This system demonstrated that pre-targeting can be leveraged for localized enrichment of therapeutics.

**Fig. 17:**
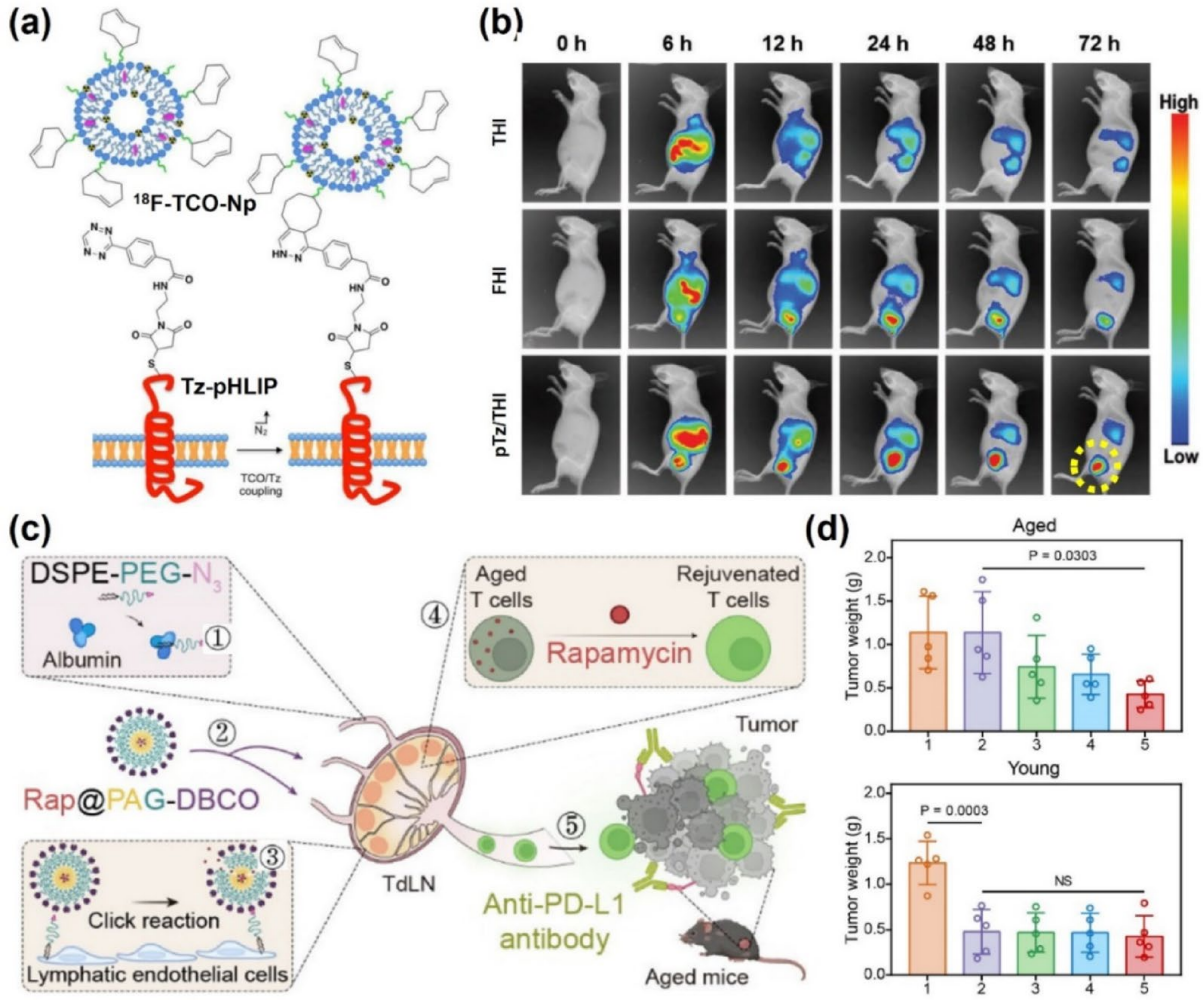
**a** Schematic representation of pHLIP-Tz-mediated pretargeting for rapid accumulation and covalent anchoring of ^18^F-TCO-liposomes in tumor tissue. Reprinted from Ref. [203] with permission from the American Chemical Society, copyright 2013. **b** Comparison of in vivo fluorescence imaging in mice following treatment with different approaches at various time points. Reprinted from Ref. [204] with permission from Wiley–VCH, copyright 2018. **c** Schematic overview of DSPE-PEG-N_3_-(DPG-N_3_) mediated pretargeting to promote the accumulation of rapamycin (Rap)-encapsulated micelles in tumor-draining lymph nodes (TdLNs). **d** Antitumor efficacy of the TdLN-targeted delivery of Rap combined with αPD-L1 therapy. Tumor weights were measured at day 28 after MC38 tumor inoculation in resected tumors from young and aged mice. 1: Control, 2: αPD-L1, 3: DPG-N_3_ + Rap + αPD-L1, 4: Rap@PAG-DBCO + αPD-L1, 5: DPG-N_3_ + Rap@PAG-DBCO + αPD-L1 Reprinted from Ref. [206] with permission from the American Chemical Society, copyright 2025

Reiner et al. demonstrated a pre-targeting strategy based on explicit in vivo anchoring [203]. Their platform combined ^18^F-labeled, TCO-functionalized liposomes (^18^F-TCO-Np) with tetrazine-conjugated pHLIP (Tz-pHLIP) which selectively labels acidic tumor cell membranes (Fig. 17a). In this case, the bioorthogonal reaction covalently immobilized the liposomes on the pre-tagged tumor surface, thereby increasing retention and enabling PET imaging with the short-lived ^18^F label. In a related approach, Xie et al. also employed tetrazine-conjugated pHLIP (pTz) for tumor-localized tagging of TCO-functionalized human serum albumin NPs loaded with the photothermal agent ICG [204]. Compared with passive accumulation (THI) or conventional folate receptor-based active targeting (FHI), the IEDDA-mediated pre-targeting strategy (pTZ/THI) markedly improved tumor localization (Fig. 17b).

Hyeon et al. reported a click reaction-assisted immune cell targeting strategy in which TCO-functionalized anti-CD11b antibodies were first administered to label inflammatory CD11b + cells, followed by tetrazine-functionalized MSNs loaded with doxorubicin [205]. In this system, in vivo tetrazine–TCO ligation enabled the NPs to hitchhike on tumor-recruited myeloid cells, thereby achieving more uniform intratumoral distribution and deeper penetration into poorly vascularized tumor regions than passive, EPR-mediated delivery. Notably, NP accumulation in vascular tumor interiors was approximately twofold higher than that achieved by conventional passive targeting, leading to enhanced therapeutic efficacy in an orthotopic 4T1 breast tumor model without evident systemic toxicity. This study is particularly noteworthy because it expanded bioorthogonal pre-targeting to the recruitment of endogenous migratory immune cells as active carriers for deep-tumor delivery of nanomedicines. More recently, Zhang et al. extended the bioorthogonal pre-targeting concept for localizing nanomedicines to tumor-draining lymph nodes (TdLNs), which are critical sites for antitumor immune priming [206]. Their platform first introduced azide groups into TdLNs via intradermal administration of DSPE-PEG-N_3_, which trafficked to the lymph nodes via albumin hitchhiking and subsequently inserted into local cell membranes (Fig. 17c). In a second step, DBCO-functionalized rapamycin-loaded PLA-PEG micelles (Rap@PAG-DBCO) were administered intradermally, enabling local bioorthogonal ligation and enhanced rapamycin enrichment within TdLNs. Functionally, this strategy rejuvenated aged CD8 + T cells and enhanced the antitumor efficacy of anti-PD-L1 therapy in aged mice to a level comparable to that observed in young animals (Fig. 17d). This work is significant because it broadens the scope of bioorthogonal pre-targeting to lymph node-directed immune modulation, highlighting its potential for immunotherapy-oriented nanomedicine.

**Fig. 18:**
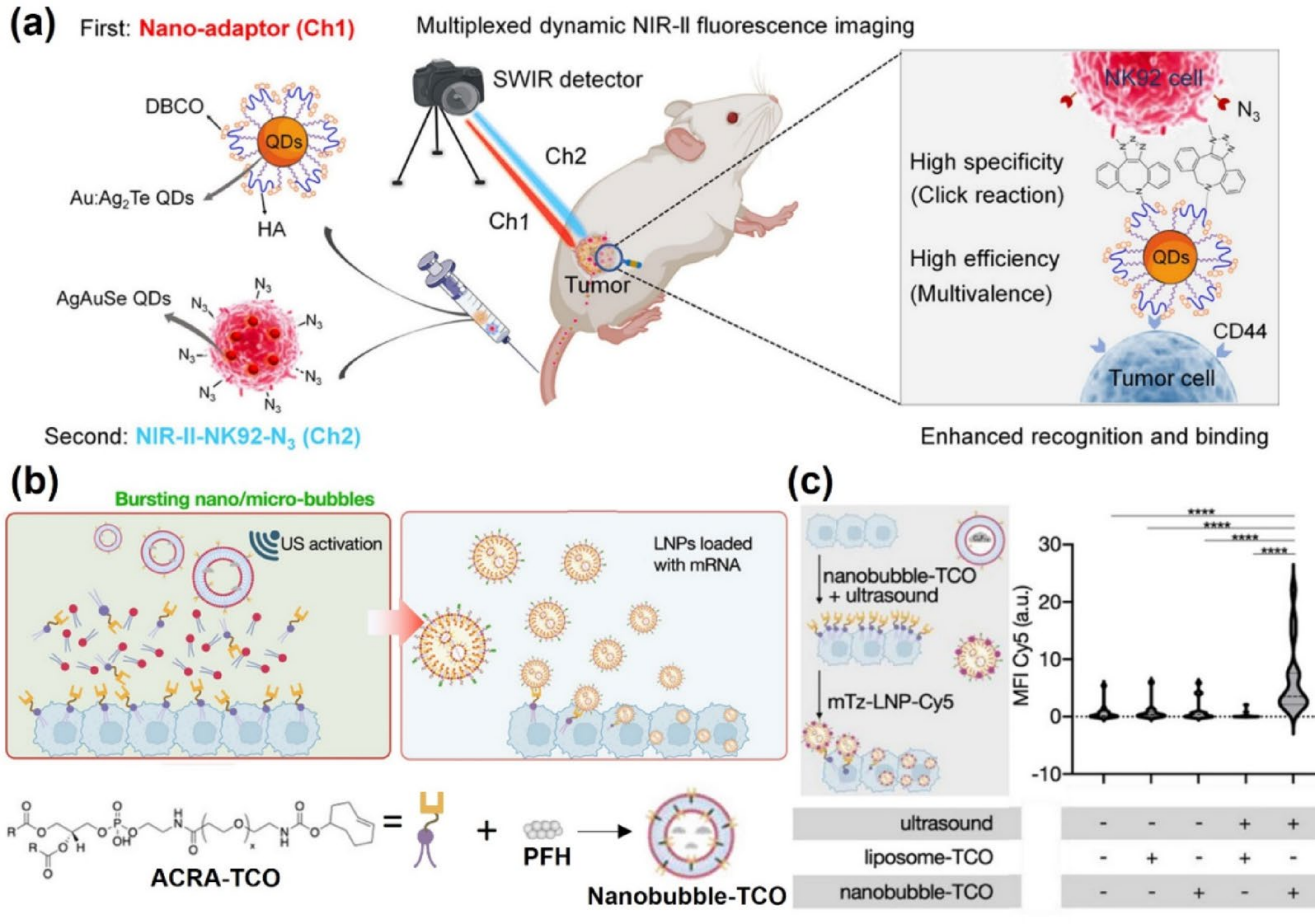
**a** Schematic representation of nanoadaptor-enabled tumor recognition by NIR-II-NK92-N_3_ cells through bioorthogonal click chemistry. Reprinted from Ref. [207] with permission from the American Chemical Society, copyright 2024. **b** Schematic illustration of a focused ultrasound-enabled bioorthogonal pretargeting strategy for targeted delivery of mRNA-LNPs using ACRA-loaded nanobubbles. **c** Comparison of intracellular Cy5 fluorescence intensity in YUMM1.7 cells after treatment with different pretargeting groups followed by mTz-LNP-Cy5. liposome-TCO: ACRA-TCO liposome without PFH, nanobubble-TCO: ACRA-TCO liposome with PFH. Reprinted from Ref. [209] with permission from Wiley–VCH, copyright 2026

Pre-targeting has also been adapted to enhance immune-cell recognition of tumors. Wang et al. reported a nanoadaptor based on hyaluronic acid (HA)/DBCO-functionalized Au:Ag_2_Te quantum dots that first accumulated in tumors through HA–CD44 recognition and then provided multivalent DBCO chemical receptors to capture azide-engineered NK92 cells (NK92-N_3_) via SPAAC chemistry (Fig. 18a) [207]. This strategy was designed to overcome a major limitation of adoptive cell therapy, namely inefficient recognition of and binding to tumor cells by therapeutic immune cells in vivo. The nanoadaptor showed progressive tumor enrichment and enabled more efficient NK-cell recruitment. In vitro, it increased NK92-mediated killing of A549 cells to 81.9%, representing a 54% enhancement over native NK92 cells; in vivo, it improved NK-cell tumor enrichment by ~ 1.9-fold relative to controls.

The pre-targeting concept has also been extended beyond oncology to nonmalignant disease imaging. Herranz et al. reported a nanoparticle-to-nanoparticle pre-targeting strategy for atherosclerosis in which TCO-functionalized sphingomyelin solid lipid NPs first accumulated in plaques, followed by administration of tetrazine- and ^68^ Ga-functionalized IONP with a short half-life (^68^ Ga, 67.8 min) as the second NP radiotracer [208]. Clear uptake in the aortic arch was observed only in the pre-targeting group, suggesting that bioorthogonal nanoparticle-to-nanoparticle detection can enable noninvasive plaque imaging without the need for conventional biological targeting ligands. This study is significant because it broadens the scope of pre-targeting from tumor imaging to NP-defined disease detection in cardiovascular pathology.

A further expansion of pre-targeting concept into nucleic acid delivery was reported by Miller et al., who developed an ultrasound-enabled bioorthogonal pre-targeting strategy for mRNA-loaded lipid nanoparticles (LNPs) [209]. Their platform employed amphiphilic click-reactive anchors (ACRA-TCO), consisting of TCO-functionalized phospholipid–PEG conjugates (Fig. 18b). In this design, ACRA-TCO was formulated as nanobubble-like liposomes encapsulating perfluorohexane (PFH), which served as a precursor for ultrasound-responsive gas generation. Administration of ACRA-TCO with focused ultrasound enabled deposition of the reactive anchors into cell membranes at the treated site. A complementary methyltetrazine (mTz)-functionalized mRNA-LNP was then administered as a second step to achieve local ligation. This ultrasound-guided pre-targeting strategy increased the fluorescence signal of the mTz-functionalized Cy5-labeled LNPs by 8- to 30-fold in cell culture (Fig. 18c) and by up to 3.6-fold in vivo, while also enhancing mRNA-mediated gene expression in vivo. Collectively, this work demonstrates that bioorthogonal pre-targeting can be integrated with physical targeting modalities such as focused ultrasound to support site-specific nucleic acid delivery in extrahepatic tissues.

### Metabolic glycoengineering-enabled artificial chemical receptors for bioorthogonal pre-targeting

**Fig. 19:**
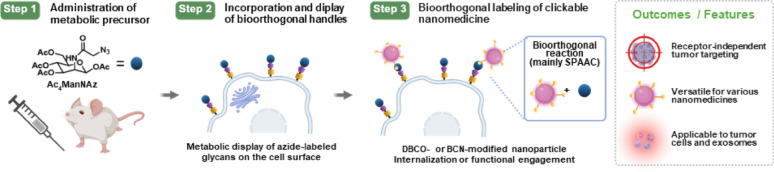
Schematic illustration of metabolic glycoengineering-enabled artificial chemical receptors for bioorthogonal pre-targeting

Despite extensive efforts to improve disease targeting by decorating nanoparticle surfaces with antibodies, receptor ligands, or small-molecule inhibitors, these strategies remain constrained by the absence of suitable receptors or by heterogeneous receptor expression within some diseased contexts [210–212]. As an alternative, MGE mediated installation of bioorthogonal handles has emerged as a strategy for introducing artificial “chemical receptors” onto target cells (Fig. 19) [213, 214]. This strategy reframe disease targeting as a programmable chemical recognition process, in which nanomedicines bearing complementary reactive groups are subsequently administered for selective in vivo ligation. In this chapter, several representative studies are discussed, highlighting the broadening biological scope of this approach (Table 5).

**Table 5:** Representative metabolic glycoengineering-enabled artificial chemical receptor systems for nanomedicine targeting

First component	Second component	Reaction type	Reaction site	Functional outcome	Refs
Ac₄ManNAz	DBCO-liposomes	SPAAC	Tumor cell surface	Tumor-selective liposome accumulation / NIRF imaging	[215]
Ac₄ManNAz-CNPs	BCN-Ce6-CNPs	SPAAC	Tumor region	Photodynamic therapy	[216]
Ac₄ManNAz-treated stem cells	BCN-CNP-Cy5.5 BCN-CNP-IRON BCN-CNP-GOLD	SPAAC	Stem cell surface	Stem cell tracking / optical, MR, CT imaging	[217]
Ac₄ManDBCO	Azido-modified silica nanoconjugates	SPAAC	Tumor cell surface	Tumor targeting / multimodal imaging	[218]
Ac₄ManNAz	POX-PCL micelles	Staudinger ligation	Azide-labeled tumor	Doxorubicin delivery / tumor-targeted chemotherapy	[219]
Ac₄ManNAz-LP	DBCO-ZnPc-LP	SPAAC	Tumor region	PA imaging / phototherapy	[220]
Ac₄ManNAz	DLQ/DZ nanocomposites	SPAAC	Breast tumor cell surface	Chemo-photothermal therapy	[221]
Ac₄ManNAz	NP-DBD	SPAAC	Tumor cell membrane	PDT/PTT/NIR-II FL imaging cGAS-STING activation	[222]
Ac₄ManNAz	EA-Pt@MDBCO	SPAAC	Orthotopic NSCLC region	Cisplatin-resistant chemotherapy / NO-assisted therapy	[223]
N₃@Gel	Ce6@DBCO-NP	SPAAC	Postoperative residual tumor region	Residual tumor PDT / antitumor immunity / metastasis inhibition	[224]
Ac₄ManNAz	^177^Lu-Fe₃O₄@HA/DBCO	SPAAC	Bladder cancer cell surface	MR imaging / radionuclide therapy / downstaging	[225]
Ac₄ManNAz	Ln-DBCO	SPAAC	Orthotopic liver tumor	Deep-tissue NIR-II imaging	[226]
Ac₄ManNAz	GBD gadolinium nanoprobe	SPAAC	Tumor cell surface	MRI-guided radiosensitization	[227]
Ac₄ManNAz-labeled cancer cells	DBCO-LNPs	SPAAC	Cancer cell surface	Cell-specific mRNA delivery	[228]
Ac₄ManNAz-DP	DBCO-GEM/ISO-DP	SPAAC	Pancreatic tumor cells / tumor-derived exosomes	Primary tumor therapy / exosome targeting / anti-metastasis	[229]

**Fig. 20:**
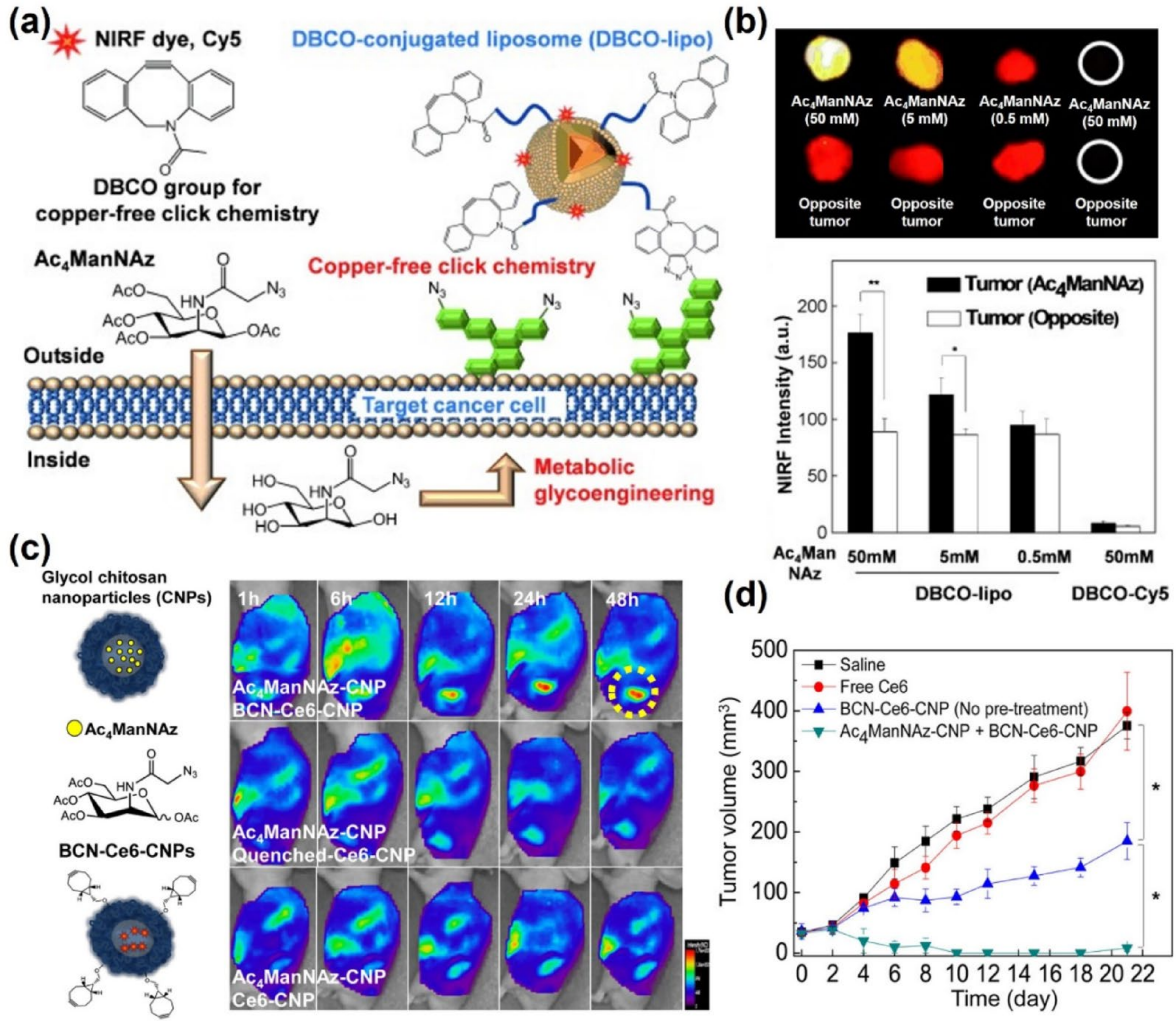
**a** Scheme of MGE-mediated tumor targeting of nanoparticles via SPAAC ligation. **b** Ex vivo NIR fluorescence images and quantified tumor signals after intravenous injection of DBCO-lipo into Ac_4_ManNAz-treated tumor-bearing mice. *p < 0.05, **p < 0.01. Reprinted from Ref. [215] with permission from Wiley–VCH, copyright 2012. **c** In vivo distribution of BCN-Ce6-CNP after Ac_4_ManNAz-CNP pretreatment, monitored by Ce6 fluorescence in the NIRF channel. Quenched-Ce6-CNP denotes the control in which the azide reactivity of BCN-Ce6-CNP was blocked. yellow circles mark tumors. (d) Tumor growth profiles monitored over 21 days after photodynamic therapy. *p < 0.01 (n = 5). Reprinted from Ref. [216] with permission from the American Chemical Society, copyright 2014

Kim et al. established the initial proof-of-concept for NP MGE-enabled bioorthogonal pre-targeting, demonstrating that intratumoral administration of Ac_4_ManNAz could metabolically introduce azido sialic acids onto tumor cells and thereby promote tumor-selective labeling by DBCO-functionalized liposomes through a SPAAC reaction (Fig. 20a) [215]. In this study, tumor accumulation of the DBCO-liposomes, monitored using Cy5-labeled liposomes, increased in an Ac_4_ManNAz dose-dependent manner and reached nearly twofold higher levels than those observed in saline-treated tumors (Fig. 20b). To improve tumor-selective MGE, the same group subsequently developed Ac_4_ManNAz-loaded glycol chitosan nanoparticles (Ac_4_ManNAz-CNPs), which were systemically administered to generate azide groups preferentially in tumors through EPR-assisted delivery and intracellular metabolic labeling, followed by treatment with BCN-modified drug-loaded glycol chitosan NPs as the second-step nanomedicine (Fig. 20c) [216]. This design addressed a key limitation of the earlier proof-of-concept by eliminating the need for local injection and demonstrating that artificial receptors could be installed in vivo through nanocarrier-mediated delivery of the metabolic precursor. The enhanced tumor accumulation of the second NP translated into remarkable tumor eradication via Ce6-mediated photodynamic therapy (Fig. 20d), although the overall performance of the system still depended on efficient precursor delivery in the first step. Later, Kim et al. adapted the same framework to stem cell imaging by using Ac_4_ManNAz-treated stem cells as target cells and BCN-modified glycol chitosan NPs carrying Cy5.5, iron oxide, or gold as complementary imaging nanoprobes [217]. In this case, the artificial receptor strategy was applied for noninvasive tracking of transplanted stem cells, and the labeled cells could be monitored optically for up to 15 days.

Cheng et al. recognized that displaying DBCO on nanomedicine surfaces can be suboptimal because of its hydrophobicity, limited display density, and difficult surface functionalization [218]. To address these limitations, they reversed the polarity of the system by developing DBCO-bearing unnatural sugars to label cancer cells directly with DBCO groups, while redesigning the nanomedicine as an azide-modified ultrasmall silica nanoconjugate. Although DBCO-bearing sugars may exhibit lower labeling efficiency than azide-labeled sugars, this reverse-handle strategy improved flexibility in NP formulation and expanded the scope for dense surface functionalization. In parallel, Jiang et al. demonstrated that the same general strategy could be implemented using a different reaction type by developing triarylphosphine-bearing polymeric micelles that targeted azide-labeled tumors through Staudinger ligation [219]. This study showed that MGE-enabled artificial receptor targeting is not restricted to SPAAC-type chemistries, but can be extended to other bioorthogonal reaction pairs when compatible nanocarrier designs are available.

This pre-targeting framework was soon integrated with therapeutic nanoplatforms. Xing et al. reported a two-step system in which Ac_4_ManNAz-loaded lipid nanomicelles first generated azide groups in tumors, followed by administration of DBCO-ZnPc-LP, a ZnPc-based nanomicellar platform for photoacoustic imaging-guided synergistic phototherapy [220]. Related MGE-based strategies were subsequently used to enhance tumor-localized accumulation of a wide range of nanomedicines, including low-molecular-weight heparin-based nanoassemblies delivering doxorubicin and ZnPc for chemo-photothermal therapy of breast cancer [221], polymer–photosensitizer nanoassemblies that elicited robust tumor inhibition together with antitumor immune responses through multifaceted phototherapeutic effects [222], polymer micelles delivering cisplatin and nitric oxide to overcome cisplatin resistance in lung cancer [223], chlorin e6-encapsulated NPs for eradication of residual tumors in postoperative regions [224], ^177^Lu-labeled iron oxide nanoprobes for bladder cancer imaging and downstaging [225], lanthanide NPs for deep tissue imaging of orthotopic liver tumors via switchable NIR-II emission [226], gadolinium NPs for one-dose MRI-guided radiosensitization [227], and lipid NPs encapsulating mRNA therapeutics for cell type-specific transfection [228].

**Fig. 21:**
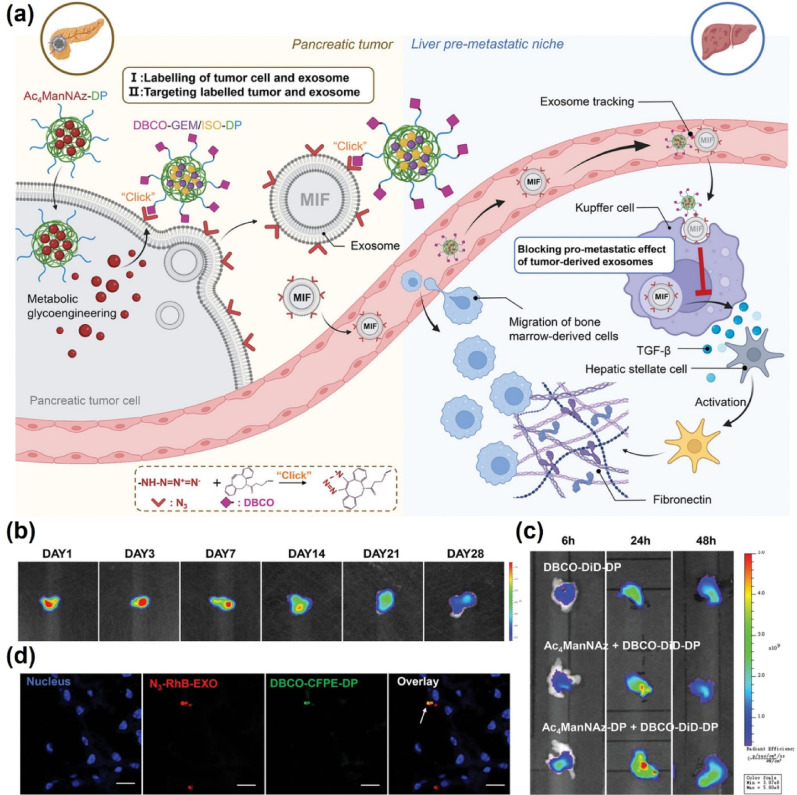
**a** Schematic overview of a sequential bioorthogonal targeting strategy for pancreatic tumors and tumor-derived exosomes (TDEs). **b** Longitudinal evaluation of azide-labeling persistence in orthotopic PAN02 tumors by IVIS imaging, using DBCO-Cy5 as a fluorescent probe for azide visualization. **c** In vivo tumor distribution of DiD-loaded DBCO nanoparticles. **d** In vivo hepatic colocalization of azide-labeled exosomes (red) and DBCO-functionalized nanoparticles (green), as indicated by the white arrows. Reprinted from Ref. [229] with permission from Wiley–VCH, copyright 2023

In another study, He et al. demonstrated that MGE-strategy could target both primary tumor cells and tumor-derived exosomes (Fig. 21a) [229]. They showed that Ac_4_ManNAz-loaded nanoparticles (Ac_4_ManNAz-DP) generated stable azide labels on pancreatic tumor cells with in vivo fluorescent ligation using DBCO-Cy5 persisting for up to 28 days (Fig. 21b). This durable metabolic labeling enabled enhanced second-step targeting, as DBCO-labeled nanoparticle carrying DiD fluorophore (DBCO-DiD-DP) exhibited the highest tumor accumulation in Ac_4_ManNAz-DP-pretreated mice (Fig. 21c). In addition, clear hepatic colocalization of azide-expressing exosomes and CFPE fluorophore labeled DBCO-DP (DBCO-CFPE-DP) confirmed that exosomes could also be targeted in vivo through bioorthogonal ligation (Fig. 21d). Therapeutically, DBCO-functionalized nanoparticles carrying chemotherapeutic drugs suppressed the primary tumor and attenuated exosome-mediated liver premetastatic niche formation. Similarly, Yu et al. showed that BCN labeling of triple-negative breast cancer cells, followed by ligation with azide-functionalized macrophage membrane-coated SERS probes, enabled SERS-based tracking of metastatic spread [230]. Together, these studies extend artificial chemical receptor engineering from primary tumor cells to tumor-derived vesicles and metastatic sites.

**Fig. 22:**
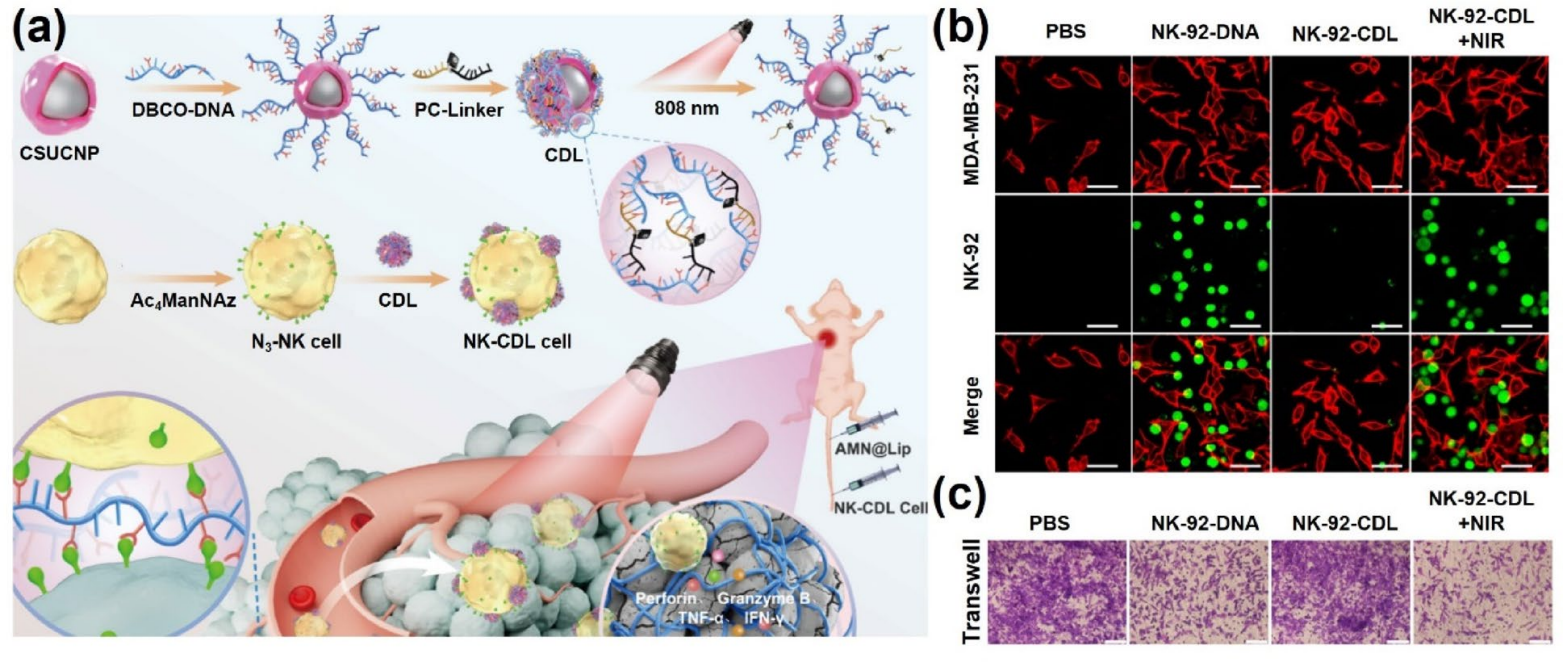
**a** Schematic overview of an NIR-responsive bioorthogonal cell-assembly platform, in which NK-CDL cells are induced under 808 nm irradiation to form cell assemblies that suppress tumor metastasis. **b** Confocal laser scanning microscopy images showing assembly formation between NK-92 cells and MDA-MB-231 cells. MDA-MB-231 cells and NK-92 cells were labeled with DiD (red) and calcein-AM (green), respectively. NK-92-DNA denotes NK-92 cells incubated with DBCO-DNA chains and served as a positive control. **c** Transwell invasion assay. MDA-MB-231 cells were stained by crystal violet. scale bar, 50 μm. Reprinted from Ref. [231] with permission from the American Chemical Society, copyright 2024

The MGE-strategy was further extended to dynamic regulation of cell–cell assembly. Liang et al. reported a NIR-activatable bioorthogonal assembly platform based on core–shell upconversion nanoparticles (CDL) decorated with long DBCO-bearing single-stranded DNA (Fig. 22a) [231]. In this design, the DNA tentacles were initially hybridized with short complementary strands containing a photosensitive linker, thereby masking most DBCO groups. After MGE introduced azides onto both NK cells and tumor cells, NK cells were administered together with CDL. Upon NIR irradiation, the DBCO-rich DNA tentacles became exposed and induced bioorthogonal assembly between immune and tumor cells. This engineered cell–cell assembly was confirmed by confocal laser scanning microscopy (Fig. 22b) and was associated with reduced tumor-cell invasion in a transwell assay (Fig. 22c), supporting its antimetastatic potential. This study extended artificial receptor engineering beyond NP homing to externally controlled multicellular assembly in vivo.

A distinct extension was reported by Fratila et al., who showed that bioorthogonal immobilization of magnetic NPs on cell membranes, followed by induction of local magnetic hyperthermia, can enable on-demand modulation of membrane permeability [232]. Under an alternating magnetic field, the membrane-bound NPs produced localized thermal perturbation at the cell surface and promoted intracellular entry of membrane-impermeable cargos, including fluorophores and siRNA, without causing overt cell death or major disruption of the cell cycle. This work broadened the scope of the artificial receptor concept beyond targeting alone and demonstrated that MGE-enabled bioorthogonal ligation can also be used to program cell-surface function and membrane permeability.

### Bioorthogonal assembly and depot formation for emergent imaging and therapy

**Fig. 23:**
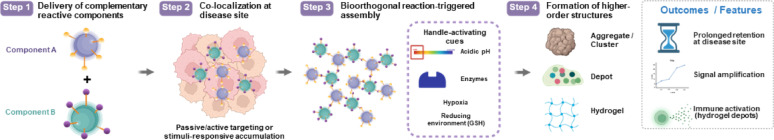
Schematic illustration of bioorthogonal assembly and depot formation for emergent imaging and therapy

Bioorthogonal chemistry can also induce in situ changes in the physical state of nanomedicine systems (Fig. 23). This chapter focuses on representative examples in which reaction-driven assembly generates emergent functions, including altered retention, enhanced imaging contrast, and new therapeutic behaviors that are not present in the initially dispersed state (Table 6).

**Table 6:** Representative bioorthogonal assembly and depot-forming systems for emergent imaging and therapy

First component	Second component	Reaction type	Reaction site	Functional outcome	Refs
DA-Cys D-NP	CBT C-NP	CBT–Cys condensation	Acidic tumor microenvironment	Intratumoral drug depot sustained extracellular drug release metastasis inhibition	[233]
iCPDN-DBCO	iCPDN-N₃	SPAAC	Acidic tumor microenvironment	On-site size transformation / DOX-NO chemoimmunotherapy	[234]
NPce6-DBCO	TK-PAMAMPR104A-N₃	SPAAC	Acidic tumor region + laser-irradiated tumor	Drug depot formation / PDT + hypoxia-activated therapy	[235]
AMD@iNP-DBCO	CPI@iNP-N₃	SPAAC	Acidic glioblastoma microenvironment	Extracellular drug depot / GBM immunotherapy	[236]
Azide-IONPs	Alkyne-IONPs	SPAAC	MMP2/9-rich CXCR4-positive tumor	T₂ MRI signal amplification	[237]
CRUN	Cathepsin B	CBT–Cys condensation	Cathepsin B-rich tumor cells / lysosome	UCL amplification / PDT / PA imaging	[238]
AuNP@1	Furin + intracellular GSH	CBT–Cys condensation	Furin-overexpressing tumor cells	Intracellular AuNP aggregation / PTT	[239]
ECN	EAD	SPAAC	Weakly acidic and hypoxic tumor	T₁–T₂ switchable MRI / accurate tumor diagnosis	[240]
N₃-DT-Exos	DBCO-bALG	SPAAC	Postoperative glioblastoma tumor bed	Exosome-cross-linked artificial lymph node gel / postoperative immunotherapy	[241]

**Fig. 24:**
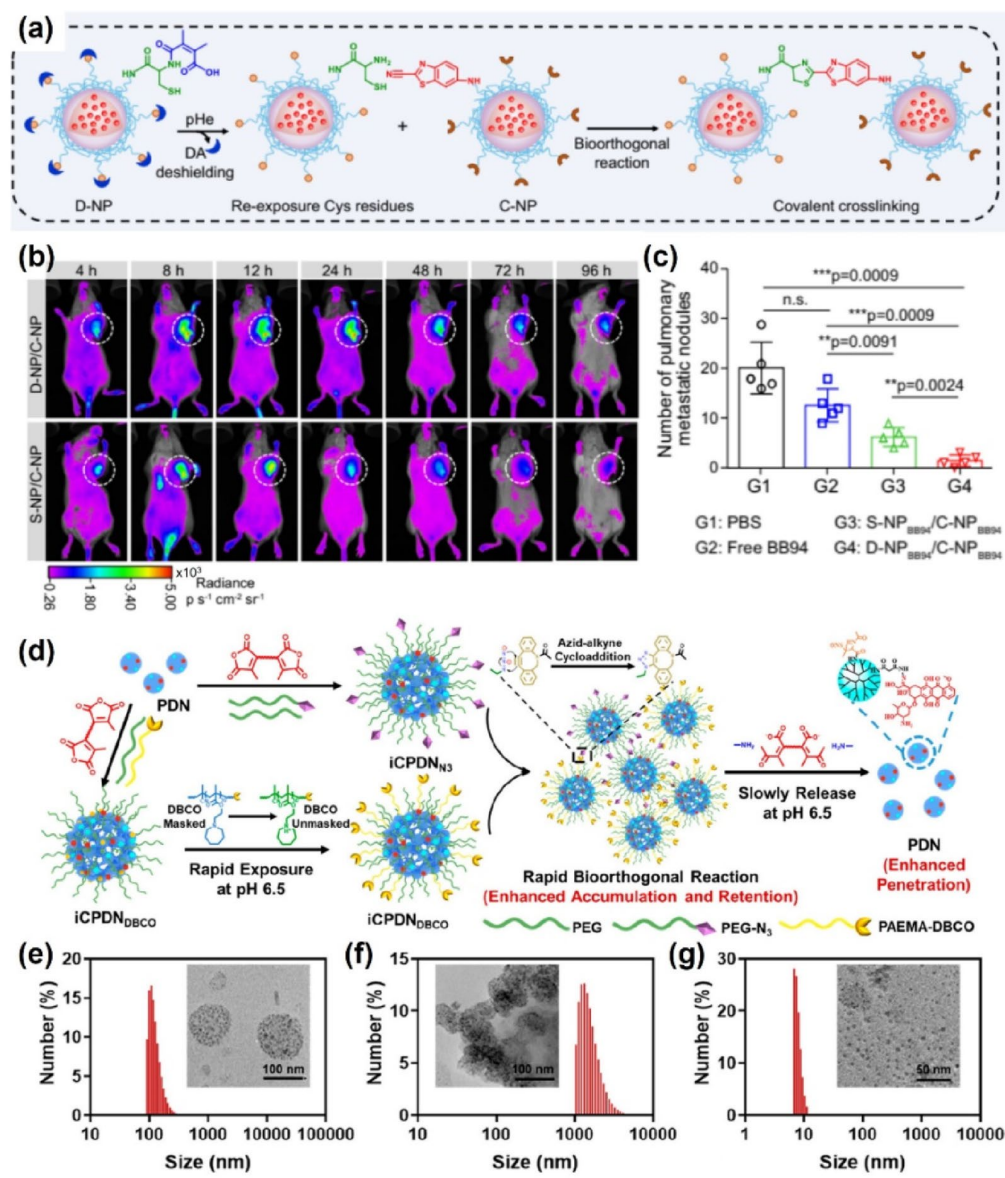
**a** Schematic representation of a bioorthogonal in situ assembly approach for generating intratumoral drug depots using two complementary nanoparticles, D-NP and C-NP. **b** In vivo images of tumor-bearing mice acquired at different time points after intravenous administration of either DiD-labeled D-NP/C-NP or S-NP/C-NP (n = 3). S-NP represents the acid-insensitive formulation as negative control for D-NP. White circles denote the tumor regions. **c** Quantification of metastatic lung nodules in tumor-bearing mice. Reprinted from Ref. [233] with permission from Springer Nature, copyright 2022. **d** Schematic overview of an acidity-responsive, bioorthogonally assembled size-transformable clustered nanosystem. **e** Hydrodynamic size of iCPDN. **f**, **g** Changes in the hydrodynamic size of iCPDN-N_3_ and iCPDN-DBCO after coincubation in pH 6.5 buffer at 37 °C for 15 min **f** and 12 h g. Insets show the corresponding TEM images. Reprinted from Ref. [234] with permission from the American Chemical Society, copyright 2022

To address a persistent limitation in nanomedicine—namely, that although many NPs extravasate into tumors, only a small fraction is retained long enough to enable efficient drug delivery at the site of action—Yang et al. exploited bioorthogonal chemistry to generate intratumoral depots that enhance local retention and improve delivery to intratumoral targets [233]. Specifically, they developed a two-NP system based on PEG-*b*-PLA block copolymers, consisting of 2,3-dimethylmaleic anhydride (DA)-masked cysteine-bearing nanoparticles (D-NP) and 2-cyanobenzothiazole (CBT)-functionalized nanoparticles (C-NP) (Fig. 24a). Under the mildly acidic conditions of the tumor microenvironment, removal of the DA mask exposed cysteine residues, which then underwent CBT–Cys condensation to crosslink adjacent NPs into microscale aggregates. The resulting in situ depots markedly prolonged tumor retention for 96 h (Fig. 24b) and were particularly effective for delivery of the extracellular matrix metalloproteinase inhibitor batimastat, reducing pulmonary metastatic nodules by 93% in an orthotopic 4T1 model (Fig. 24c).

Yuan et al. extended this extracellular assembly strategy to a dynamic, in situ size-transformable platform designed to balance tumor retention with deep tissue penetration under hypoxic conditions [234]. Their platform employed two acidity-responsive dendritic poly(amidoamine) (PAMAM)-based nanosystems, iCPDN-DBCO and iCPDN-N_3_, in which the DBCO groups were initially masked but were exposed in the acidic tumor microenvironment (Fig. 24d). The initial hydrodynamic diameter of iCPDN was approximately 102.9 nm; however, exposure of DBCO enabled rapid reaction with complementary azides, producing large aggregates of about 1216 nm within 15 min (Fig. 24e, f). These aggregates increased tumor accumulation and prolonged retention, whereas a second, more slowly cleavable acid-responsive motif subsequently released ultrasmall PAMAM-DOX/NO (PDN) units (~ 10 nm) after 12 h (Fig. 24g), which were better suited for deep tumor penetration. Functionally, this design enabled efficient co-delivery of doxorubicin and nitric oxide, with the latter alleviating hypoxia-induced chemoresistance by downregulating HIF-1α. Thus, the aggregate-to-small-particle transition provided an effective means to integrate prolonged tumor retention with improved intratumoral penetration. The same group later reported a light-responsive, size-transformable nanomedicine in which acid-triggered bioorthogonal crosslinking first generated a perivascular depot, followed by ROS-mediated linker cleavage under laser irradiation to release smaller therapeutic nanoparticles for deeper penetration into hypoxic tumor regions [235]. This design combined bioorthogonal assembly with stimulus-triggered disassembly, enabling spatiotemporally controlled redistribution of therapeutics within the tumor. The similar design principle has recently been extended to immunotherapy for glioblastoma [236]. In this study, acidity-responsive AMD3100-loaded micelles (AMD@iNP-DBCO) and CPI-444-loaded micelles (CPI@iNP-N_3_) underwent in situ extracellular assembly after acid-triggered exposure of DBCO in the tumor microenvironment, thereby forming large local depots. Because both therapeutics, AMD3100 and CPI-444, act primarily within the extracellular or membrane-proximal immune milieu, this strategy enhanced local drug release to modulate the tumor immune response.

**Fig. 25:**
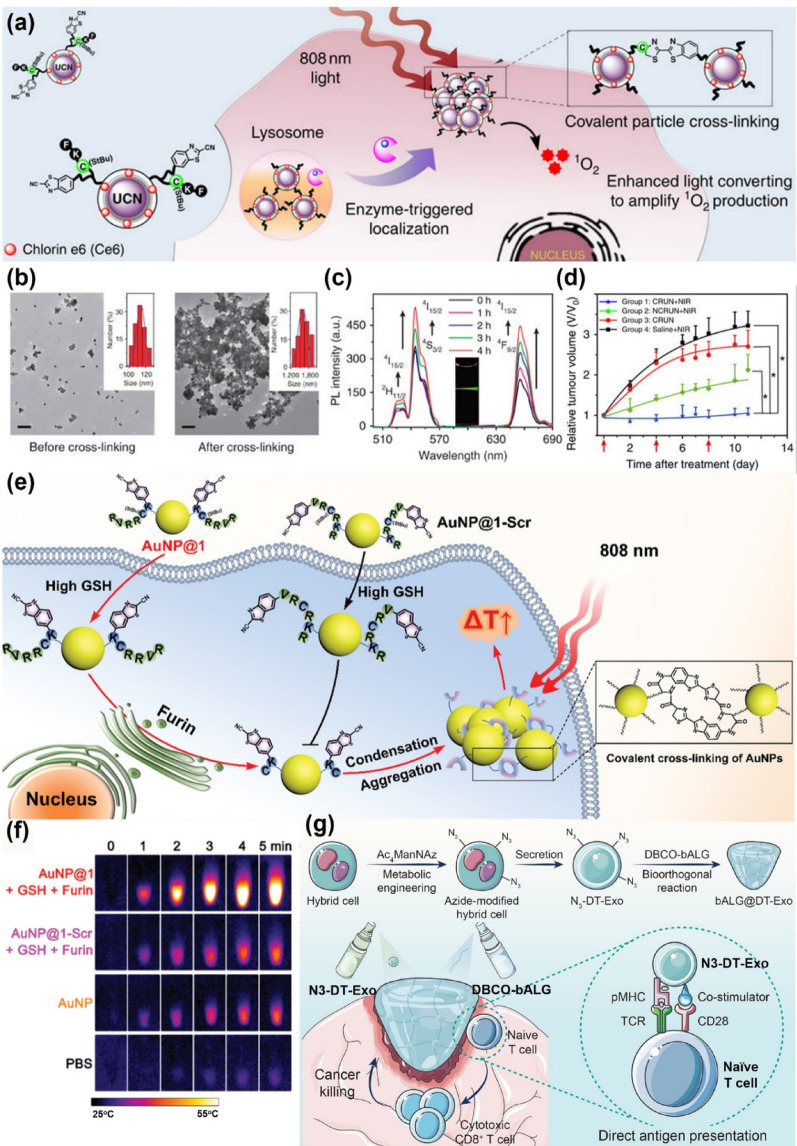
**a** Schematic illustration of lysosomal cathepsin B-mediated assembly of CRUN through the bioorthogonal CBT–Cys condensation reaction. **b** TEM images and DLS measurements of CRUN in the absence (left) or presence (right) of cathepsin B. **c** Upconversion luminescence of CRUN measured at different time points after cathepsin B treatment. The inset shows a luminescence image of CRUN. **d** Changes in relative tumor volume in the indicated treatment groups following photodynamic therapy under 808 nm laser irradiation. Reprinted from Ref. [238] with permission from Springer Nature, copyright 2016. (e) Schematic representation of furin-guided intracellular aggregation of AuNP@1 through CBT–Cys condensation. **f** Infrared thermal images of each group, recorded during 808 nm NIR laser irradiation (2 W/cm^2^, 5 min). Reprinted from Ref. [239] with permission from Wiley–VCH, copyright 2020. **g** Schematic illustration of N3-DT-Exo preparation and the in situ formation of immunotherapeutic gels in postoperative glioblastoma lesions via the SPAAC reaction, Reprinted from Ref. [241] with permission from the American Chemical Society, copyright 2024

An early example of aggregation-enabled functional amplification was reported by Long et al., who developed CXCR4-targeted and MMP-responsive iron oxide NPs for enhanced MRI [237]. In this system, two complementary iron oxide NP populations bearing masked azide and cyclooctyne functionalities were delivered to CXCR4-positive tumors, where MMP2/9-mediated peptide cleavage exposed the reactive handles and enabled SPAAC chemistry. The resulting self-assembled magnetic nanoclusters increased local iron accumulation and amplified T2 contrast in vitro and in vivo, demonstrating that tumor-selective bioorthogonal assembly can generate emergent imaging enhancement through cluster formation. In parallel, Xing et al. developed cathepsin B-responsive upconversion nanoconjugates (UCN) for tumor-localized imaging and photodynamic therapy [238]. In this system, peptide/Ce6-functionalized Nd^3+^-sensitized upconversion nanocrystals (CRUN) underwent cysteine–CBT-mediated covalent crosslinking after exposure of cysteine residues via cathepsin B activity, leading to localized particle aggregation within tumor cells and tumor tissue (Fig. 25a). This enzyme-responsive crosslinking induced marked particle assembly (Fig. 25b) and under 808 nm irradiation, enhanced upconversion luminescence at around 660 nm was observed (Fig. 25c), which in turn amplified singlet oxygen generation from surface-bound chlorin e6 and improved photodynamic efficacy relative to noncrosslinking controls (Fig. 25d). In another study, Wang et al. developed a furin-responsive 5 nm AuNP platform (AuNP@1) to address the trade-off between the favorable in vivo pharmacokinetics of ultrasmall AuNPs and their limited photothermal performance (Fig. 25e) [239]. Following intracellular uptake, GSH reduction and furin cleavage exposed cysteine groups that reacted with CBT to induce interparticle crosslinking and intracellular AuNP aggregation. This process increased particle size to approximately 100 nm, enhanced absorption at 808 nm, and improved photothermal heating relative to a furin-nonresponsive control (Fig. 25f). Accordingly, AuNP@1 exhibited superior photothermal therapeutic efficacy against MDA-MB-468 tumors both in vitro and in vivo.

Bioorthogonal clustering has also been exploited to generate switchable imaging contrast. Pei et al. developed a dual-key-and-lock MRI probe (DKL-CA) based on extremely small iron oxide nanoparticles (ESIONPs), in which complementary azide- and DBCO-bearing particles were masked by acid- and hypoxia-responsive PEG shells, respectively [240]. Only under the combined conditions of weak acidity and hypoxia were both shielding layers removed, enabling azide–DBCO click-induced aggregation and conversion of NPs from a dispersed T1 state to an aggregated T2 state. This strategy is notable because it uses bioorthogonal assembly to create an emergent imaging-mode switch, thereby enabling more precise tumor diagnosis with fewer false-positive signals than single-trigger systems.

In a distinct approach, Zhang et al. used bioorthogonal chemistry to form an exosome-cross-linked gel in situ that acted as an artificial lymph-node-like structure after glioblastoma surgery [241]. In this system, azide-modified exosomes (N_3_-DT-Exos) derived from dendritic cell–tumor hybrid cells were cross-linked with DBCO-modified alginate (DBCO-bALG) via SPAAC ligation to form a sprayable gel in the postoperative tumor bed (Fig. 25g). The gel preserved antigen presentation and costimulatory signals while prolonging local immune activation. This study is notable because it provides a clear example in which bioorthogonal chemistry generates an emergent, tissue-like immunological microenvironment rather than simply forming a particle cluster.

Collectively, the studies discussed in this section demonstrate that bioorthogonal chemistry can program nanomedicine behavior beyond individual nanoparticles by engineering interactions among nanocarriers, cells, and diseased tissues in vivo. Pre-targeting strategies separate slow disease-site localization from subsequent effector delivery, thereby improving spatial selectivity and accommodating pharmacokinetic mismatches between targeting carriers and short-lived or potent therapeutic or imaging agents. However, their translation requires careful coordination of dosing sequence, reaction kinetics, tissue accessibility, effector lifetime, and clearance. Metabolic glycoengineering creates artificial chemical receptors that can reduce dependence on endogenous receptor expression, but precursor specificity, labeling density, glycan turnover, off-target labeling, and membrane-ligation-induced cellular responses should be considered. In particular, the time scale of membrane ligation and internalization should be aligned with the intended site of payload action, because intracellular therapeutics such as RNA drugs require uptake and cytosolic availability, whereas extracellular or immunomodulatory agents may need to remain accessible at the cell surface to regulate immune-cell interactions. Bioorthogonal assembly and depot-forming systems can enhance retention, signal amplification, and sustained activity through trigger-gated local aggregation after tissue accumulation, but the precision of this gating process must be validated in terms of tissue penetration, uncaging kinetics, assembly kinetics, biodegradability, and long-term safety. Future studies that systematically address these design parameters will be essential for advancing higher-order bioorthogonal nanomedicine toward programmable, precise, and clinically relevant applications.

## Conclusions

Bioorthogonal chemistry is increasingly serving as a powerful design principle for nanomedicine. Across the studies discussed in this review, its role extends from modular surface functionalization to conditional payload activation and further to tissue-localized assembly, allowing NP functions to be installed or switched on after formulation, delivery, or disease-site accumulation. At the surface level, bioorthogonal reactions enable the installation of targeting ligands, imaging probes, polymers, antibodies, and biomimetic membrane components on diverse nanomaterial platforms under mild conditions while preserving structural integrity and biological activity. At the molecular level, click-to-release and related activation strategies offer a means to control when and where therapeutic payloads become pharmacologically active, rather than relying solely on passive leakage, carrier degradation, or endogenous triggers with limited selectivity. Beyond individual NPs, bioorthogonal chemistry has also enabled pre-targeting, localized depot formation, and NP–cell coupling, generating emergent functions such as prolonged retention, signal amplification, and disease-site-restricted therapy.

Despite these advances, the translational potential of bioorthogonal nanomedicine will depend on whether reaction performance can be maintained under biologically and clinically relevant conditions. In vivo reaction efficiency is governed not only by intrinsic rate constants, but also by local reactant concentration, tissue penetration, serum stability, bioavailability, dosing interval, and clearance. This is particularly important for two- or multi-component systems, where therapeutic output requires spatial and temporal coordination of multiple reactive components. Therefore, reported in vivo ligation, uncaging, or assembly efficiencies should be evaluated against clinically relevant dilution, pharmacokinetic, and dosing constraints rather than only proof-of-concept activation. For metabolic glycoengineering and cell-surface ligation strategies, precursor specificity, labeling density, glycan turnover, off-target labeling, and membrane-associated biological responses require careful assessment. For assembly- and depot-forming systems, local aggregation must be balanced with biodegradability, reversibility, clearance, and long-term safety. Future progress will require quantitative integration of reaction kinetics, nanocarrier pharmacokinetics, tissue accessibility, and biological safety. Such efforts will be essential for advancing bioorthogonal chemistry from a powerful experimental tool toward a programmable, precise, and clinically translatable strategy for nanomedicine.

## Data Availability

The review is based on the published data and sources of data upon which conclusions have been drawn can be found in the reference list.
